# Ligand Clouds around Protein Clouds: A Scenario of Ligand Binding with Intrinsically Disordered Proteins

**DOI:** 10.1371/journal.pcbi.1003249

**Published:** 2013-10-03

**Authors:** Fan Jin, Chen Yu, Luhua Lai, Zhirong Liu

**Affiliations:** 1 BNLMS, State Key Laboratory for Structural Chemistry of Unstable and Stable Species, College of Chemistry and Molecular Engineering, Peking University, Beijing, China; 2 Center for Quantitative Biology, Peking University, Beijing, China; 3 Peking-Tsinghua Center for Life Sciences, Peking University, Beijing, China; University of Uppsala, Sweden

## Abstract

Intrinsically disordered proteins (IDPs) were found to be widely associated with human diseases and may serve as potential drug design targets. However, drug design targeting IDPs is still in the very early stages. Progress in drug design is usually achieved using experimental screening; however, the structural disorder of IDPs makes it difficult to characterize their interaction with ligands using experiments alone. To better understand the structure of IDPs and their interactions with small molecule ligands, we performed extensive simulations on the c-Myc_370–409_ peptide and its binding to a reported small molecule inhibitor, ligand 10074-A4. We found that the conformational space of the apo c-Myc_370–409_ peptide was rather dispersed and that the conformations of the peptide were stabilized mainly by charge interactions and hydrogen bonds. Under the binding of the ligand, c-Myc_370–409_ remained disordered. The ligand was found to bind to c-Myc_370–409_ at different sites along the chain and behaved like a ‘ligand cloud’. In contrast to ligand binding to more rigid target proteins that usually results in a dominant bound structure, ligand binding to IDPs may better be described as ligand clouds around protein clouds. Nevertheless, the binding of the ligand and a non-ligand to the c-Myc_370–409_ target could be clearly distinguished. The present study provides insights that will help improve rational drug design that targets IDPs.

## Author Summary

Intrinsically disordered proteins (IDPs) exist as conformational ensembles that change rapidly. They are an important and common class of proteins in all kingdoms of life. IDPs are widely associated with human diseases and may serve as potential drug design targets. However, drug design targeting IDPs is difficult and only limited examples have been reported. One example is the oncoprotein, c-Myc, for which seven inhibitors were discovered by experimental screening. Understanding how small inhibitor molecules bind to c-Myc may help in understanding the binding mechanism of IDPs with ligands. In the present study, we conducted extensive molecular dynamics simulations to explore the binding mechanism for the c-Myc peptide with an inhibitor 10074-A4. We found that 10074-A4 could bind to c-Myc_370–409_ at different sites along the peptide chain and its binding behavior could be described as a ‘ligand cloud’. Even in the bound state, the structure of the c-Myc_370–409_ peptide remained a dynamic ensemble. Compared to c-Myc peptides that do not bind to 10074-A4, c-Myc_370–409_ binds selectively with 10074-A4, but the specificity of binding was not high. The interactions of IDPs with ligands can perhaps be described as a scenario in which ligand clouds around protein clouds.

## Introduction

Intrinsically disordered proteins (IDPs), discovered in the 1990s, are proteins that lack a stable three-dimensional native structure under physiological conditions [1]–[5]. IDPs are sometimes described as “protein clouds” because of their structural flexibility and dynamic conformation ensemble [6]. Various bioinformatics methods have been developed to predict IDPs based on their sequences [7], [8]. It was revealed that IDPs are abundant in all kingdoms of life; for example, more than 40% of the proteins in eukaryotic cells possess disordered regions longer than 50 residues [9], [10]. Because of the flexibility of the chain and the resulting advantages in protein-protein interactions [1], [11], [12], IDPs play important roles in various critical physiological processes such as the regulation of transcription and translation [2], cellular signal transmission, protein phosphorylation and molecular assemblies [3], [13], [14]. On the other hand, IDPs also have some adverse effects. It was revealed that many IDPs are associated with human diseases such as cancer, cardiovascular disease, amyloidosis, neurodegenerative diseases, and diabetes [15]. It was also reported that the Swiss-Prot keywords for eleven severe diseases are strongly correlated with IDPs [16]. Given their abundance and their biological importance, IDPs are regarded as promising and potential drug targets [15], [17]–[19].

Compared with rational drug design targeting ordered proteins [20]–[22], drug design targeting IDPs is still in its infancy. Though some general strategies have been proposed [23], most of the studies [24]–[30] have been limited to only a few systems, namely, p53-MDM2, EWS-FLI1 and c-Myc-Max. Among them, the oncoprotein c-Myc is an encouraging example. C-Myc is a transcription factor with a basic helix-loop-helix leucine zipper (bHLHZip) domain which becomes active by forming a dimer with its partner protein Max [31]. In their unbound forms, both c-Myc and Max are disordered. However, in the dimerized forms, they undergo coupled folding and binding. In most cancers cells, c-Myc protein is expressed persistently by a mutated *Myc* gene, causing its unregulated expression in cell proliferation and signal transmission. Therefore, inhibiting either the overexpression of c-Myc and/or its dimerization with Max may provide a therapy for cancer. Yin et al. [30] have used high-throughput experimental screening to successfully identify seven compounds that inhibit dimerization between c-Myc and Max. Further biophysical studies using nuclear magnetic resonance (NMR), circular dichroism (CD) and fluorescence assays have verified three different binding sites (residues 366–375, 374–385, and 402–409) in the bHLHZip domain of c-Myc [28]. These binding sites contain several successive residues that can independently bind different small molecules [28]–[30]. It should be noted that, after binding with the small molecule inhibitors, the c-Myc sequence remains disordered, making the detailed experimental characterization of the molecular interactions almost impossible. Therefore, the inhibition mechanism is still unclear. For example, a recent study using drift-time ion mobility mass spectrometry suggested that the binding between c-Myc and these inhibitors is not as specific as previously thought [32]. The lack of conformation data also hampers the application of the well-developed structure-based drug design approach to optimize the inhibition.

Molecular simulations are useful in understanding the characteristics of IDPs because they can provide an atomic description of molecular interactions. Coarse-grained models [11], [33]–[35] and all-atom simulation [36]–[42] have both been used to investigate IDPs. Recently, Knott and Best [40] used large-scale replica exchange molecular dynamics (REMD) simulations with a well-parameterized force field to obtain a conformational ensemble of the nuclear coactivator binding domain of the transcriptional coactivator CBP. Their simulation results were in good agreement with NMR and small-angle X-ray scattering measurements, validating the efficacy of all-atom simulations in exploring the highly dynamic conformations of IDPs. For the c-Myc/inhibitor complex described above, Michel and Cuchillo [43] built a structural ensemble using all-atom simulations for c-Myc_402–412_ with and without an inhibitor (10058-F4) and found that 10058-F4 bound to multiple distinct binding sites and interacted with c-Myc_402–412_. However, because the c-Myc segment used in their simulation contained only the 11 residues that covered the binding sites of 10058-F4 (residues 402–409), it is unclear how the inhibitors would interact with longer segments of c-Myc and how specific the interaction would be.

In the present study, we conducted extensive all-atom molecular dynamic (MD) simulations to investigate the c-Myc_370–409_ conformational ensemble and its interactions with a small-molecule inhibitor (10074-A4). First, we performed implicit-solvent REMD simulations to clarify the conformational features of the unbound c-Myc_370–409_. Next, we performed MD simulations with an explicit water model to explore in detail the interactions between c-Myc_370–409_ and 10074-A4. Finally, a negative control using a different peptide segment (c-Myc_410–437_) was simulated to address the issue of interaction specificity. The conformational ensemble that we obtained will be useful not only in clarifying the structural features of c-Myc and the binding mechanism with inhibitors, but also in providing reference structures for drug design targeting c-Myc via structure-based approaches.

## Results

### Conformational analysis of c-Myc_370–409_

Conformational sampling of IDPs for molecular modeling is challenging because the energy landscapes of IDPs are relatively flat [44], [45]. In the present study, extensive REMD simulations using an implicit solvent model were performed to explore the conformational characteristics of c-Myc_370–409_. The accumulative total of simulation time reached 34.5 µs (see Methods). C-Myc_370–409_ is a 40-residue truncated construct of a full-length c-Myc. The conformational properties of c-Myc_370–409_ in its bound state (with 10074-A4) and more dynamic unbound state, have been studied experimentally using CD and NMR spectroscopy, and a likely average conformation was built based on chemical shift data which is not meant to (and cannot) define detailed structural features [28]. We compared our simulation results with the available experimental results.

**Figure 1:**
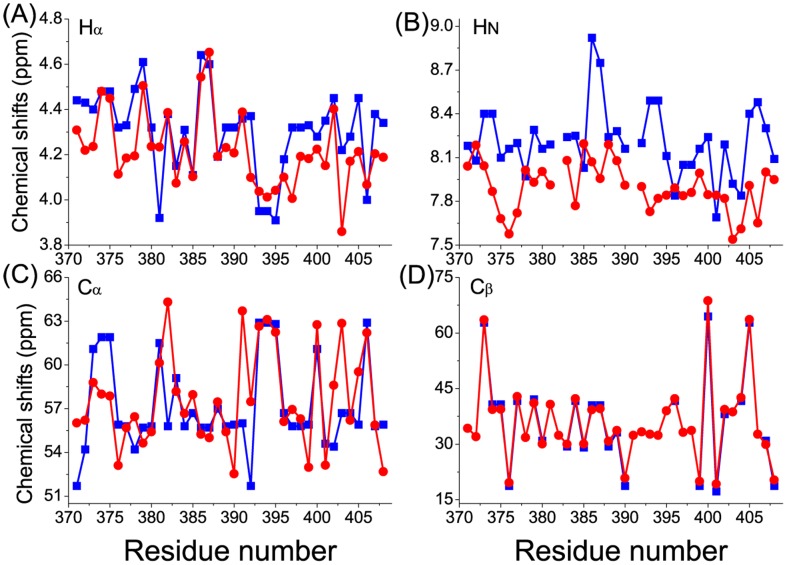
Comparisons of the computed and experimental chemical shifts for apo c-Myc_370–409_. The computed values are from the REMD simulations (red circles) and the experimental values are from Hammoudeh et al. [28] (blue squares). Note that the experimental values for some residues were not available. Chemical shifts are for the atoms: **A** H_α_, **B** H_N_, **C** C_α_, **D** C_β_.

**Figure 2:**
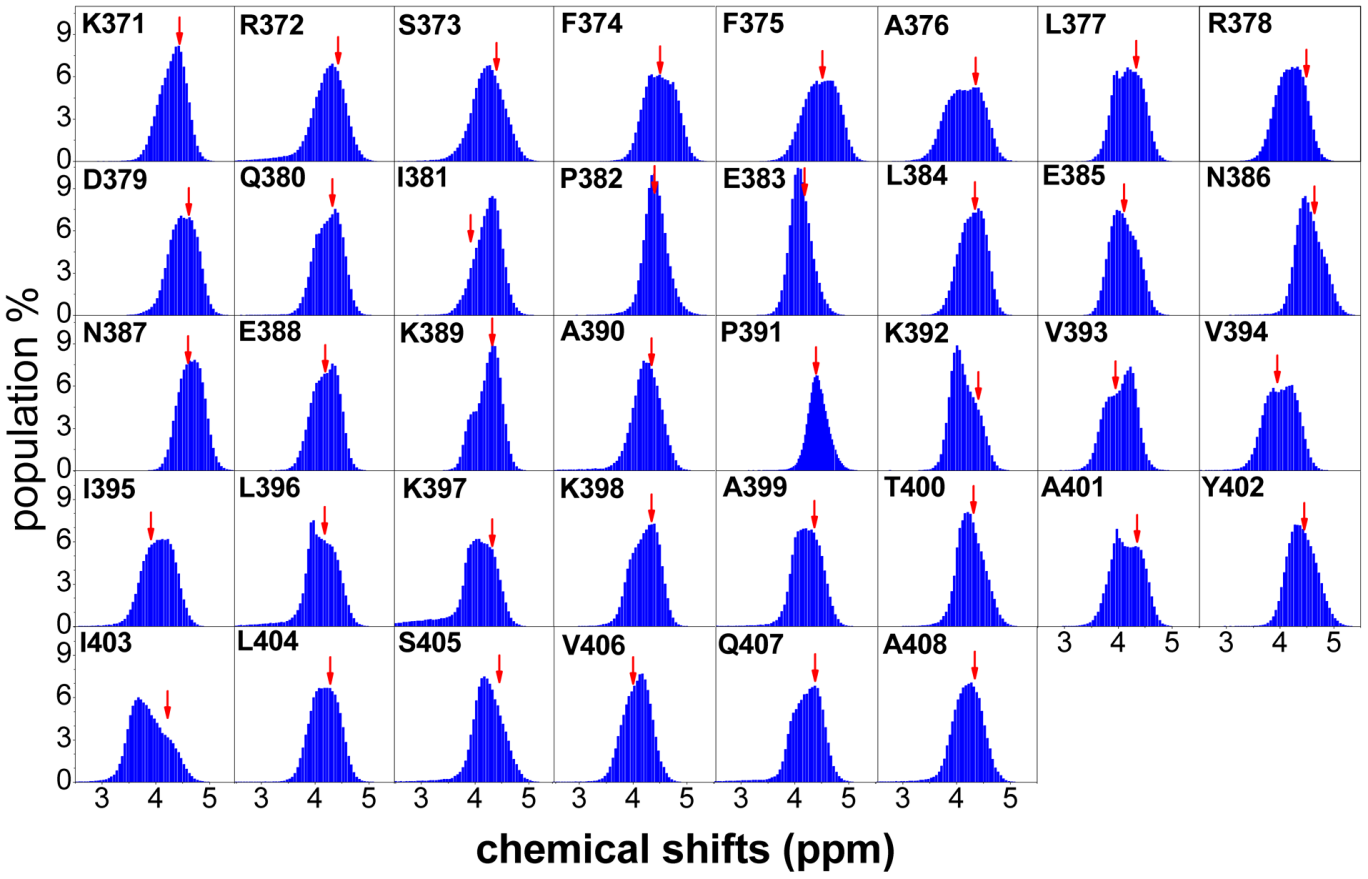
Distribution of the H_α_ chemical shifts for apo c-Myc_370–409_ determined from REMD simulations. For comparison, the experimental values are indicated by red arrows.

To assess the sampling quality of the REMD simulations, we computed ^1^H and ^13^C chemical shifts from the simulated conformational ensemble using SHIFTS [46] and compared the computed values with the experiment values (Figure 1). The agreement is reasonable, though not excellent. Deviations between the average chemical shift values for a simulated ensemble and experimental values have been observed previously in several studies on IDPs [40], [47], [48]. The chemical shift calculation performed using several other software (SHIFTX [49], CamShift [50], SPARTA+ [51]) also showed deviations between the computed and experimental values (Figure S1). A possible reason is that chemical shifts are difficult to calculate accurately and the underlying parameterizations applied in current software for the calculation of chemical shifts have been optimized for ordered proteins but not for IDPs [47]. Interestingly, when we back-calculated chemical shifts from the NMR-refined structure using either the SHIFTS [46] or SHIFTX [49] software, the resulting values also deviated from the experimental ones (Figure S2). In addition, the ensemble nature of IDP conformations suggests that the chemical shifts of IDPs should be described as a distribution, and not merely as average values. The calculated distributions of the H_α_ chemical shifts obtained from our simulations are summarized in Figure 2. All the H_α_ chemical shifts are distributed over a broad range. The experimental values, indicated by arrows in Figure 2, are located close to the centers of the distributions, indicating the validity of the conformational sampling. Data for the H_N_, C_α_ and C_β_ chemical shifts are given in Figure S3, showing similar behaviors as the H_α_ chemical shifts. We also computed the distribution of the backbone dihedral angles (Ramachandran (φ,ψ) plot) for the simulations and the dihedral angles of the NMR-refined apo structure lie well within the simulation distributions (Figure S4).

**Figure 3:**
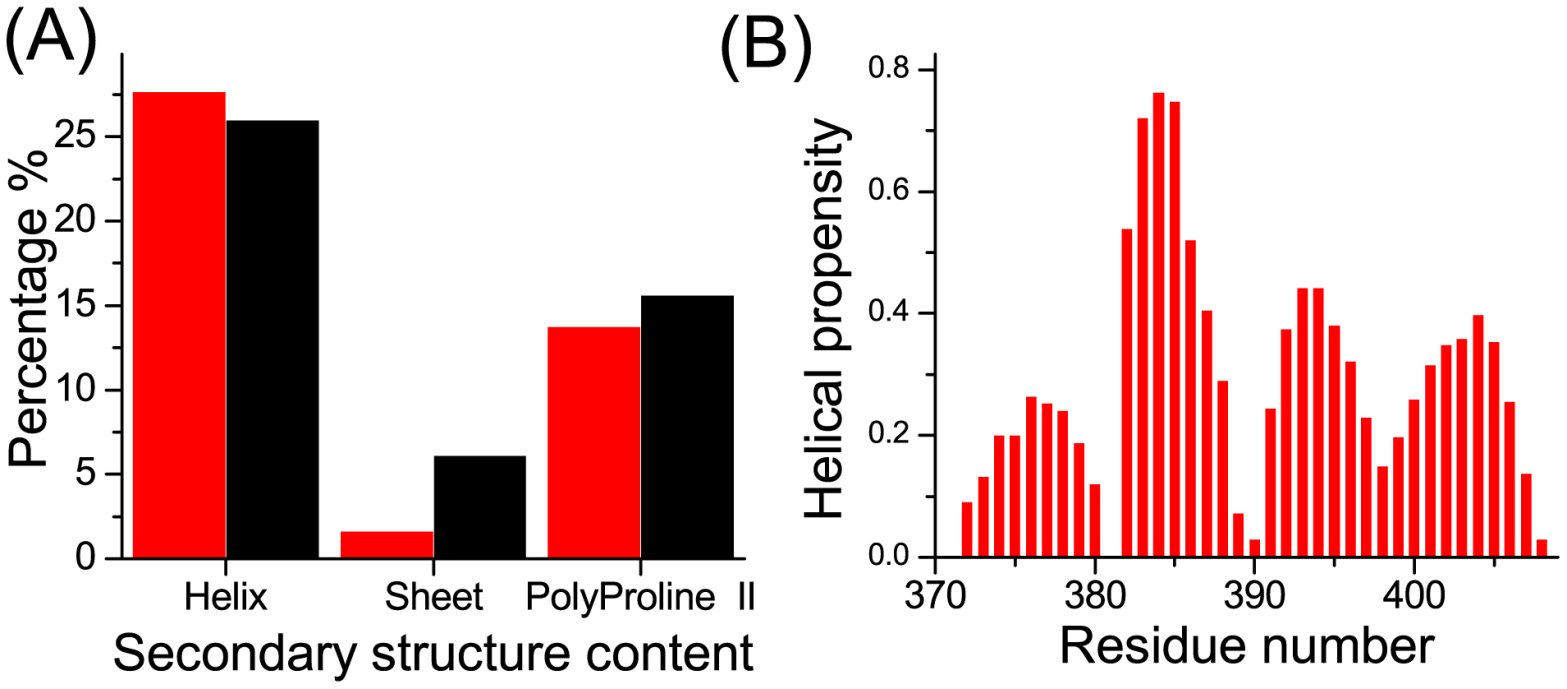
Secondary structure content and helix propensity of apo c-Myc_370–409_. **A** Secondary structure content. For the REMD simulations (red), the helix and sheet content was computed using the DSSP [52] method; the polyproline II content was computed with the PROSS software [53]. For the experimental data (black), the secondary structure content was estimated from the chemical shifts using δ2D [54]. **B** Helix propensity from the REMD simulations using the DSSP method.

The secondary structure content of the simulated structures was also calculated [43], [52]–[54] and compared with that estimated from the experimental chemical shifts (Figure 3). The helix and polyproline II content of the simulated structure were consistent with the experimental structures (Figure 3A). However, the sheet content of the simulated structures was much lower than the sheet content of the experimental structures. In a previous study [43] on a shorter c-Myc segment, c-Myc_402–412_, a similar underestimation of sheet content was observed in the simulated structures. The deficiency of sheet content in the simulated structures might be caused by a bias in the force fields. Although c-Myc_370–409_ is intrinsically disordered, it possesses a high content of residual helical structure (>25%). The simulated helix propensity (Figure 3B) showed three helical regions separated by proline residues, Pro382 and Pro391.

**Figure 4:**
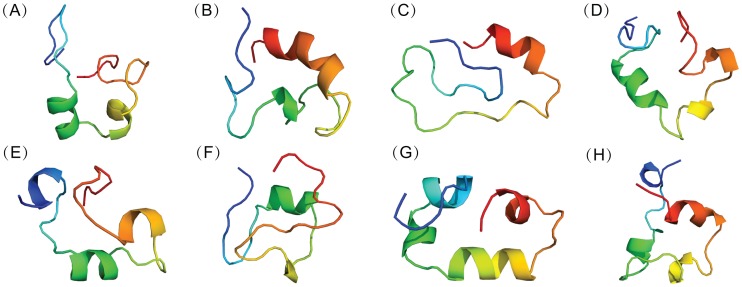
Representative conformations of apo c-Myc_370–409_ computed from REMD simulations. Backbone-RMSD clustering with a cutoff of 2.0 Å of all the conformations was performed. Representative c-Myc_370–409_ structures (from blue at the N-terminal to red at the C-terminal) for the first eight clustering groups were displayed in cartoon. The fractional cluster populations are: **A** 9.5%, **B** 8.4%, **C** 7.3%, **D** 7.1%, **E** 5.8%, **F** 5.1%, **G** 4.8%, **H** 4.1%.

To clarify the conformational features of c-Myc_370–409_, backbone-RMSD clustering with a cutoff of 2.0 Å of the conformations was performed. Representative structures (the central structure of each group) of the first eight groups were depicted in Figure 4. They are all somewhat collapsed compared to the fully extended structure and possess a rich residual helical structure. These states with considerable population will be useful references for rational drug design targeting c-Myc. The existence of residual structure may be related to the functional misfolding that prevents IDPs from unwanted interactions with non-native partners [55]. A quantitative analysis on the distributions of dimension and helix content was provided in Figure S5. The mean radius of gyration is around 10.3±0.6 Å, which is much smaller than the expected value of random coils (18.5 Å) under the same chain length. The mean helix content of the conformational ensemble is 27.7±11.1%, showing a broad distribution. These results indicated that c-Myc_370–409_ is disordered in nature and interconversions between dispersed structures occur.

### Stabilizing interactions in c-Myc_370–409_

To reveal how the conformations of apo c-Myc_370–409_ were stabilized, we analyzed the Lennard-Jones and electrostatic residue-residue interactions among all the residues (Figure S6). The Lennard-Jones interaction matrix was rather weak (Figure S6A), indicating that the conformations were disordered and that the packing in the collapsed structures was poor. This finding is consistent with the contact map, which showed that residue-residue contacts were dispersed and low in magnitude (Figure S6B). The electrostatic interactions, on the other hand, were comparatively strong (Figure S6C), probably because nearly one-third of the residues in c-Myc_370–409_ (12 out of the 40) are charged residues. The favorable electrostatic interactions of the Arg372, Arg378, Lys389, Lys392 and Lys398 residues with the Asp379, Glu383, Glu385 and Glu409 residues (Figure S6C) are the result of the electrostatic attraction between residues with opposite charges. Residues like Ser373 and Gln380 also contributed to the electrostatic interactions by forming hydrogen bonds (Figure S6D). Therefore, charge-pair interactions and hydrogen bonds were the main stabilized factors for the c-Myc_370–409_ conformations.

### Binding of 10074-A4 to c-Myc_370–409_

**Figure 5:**
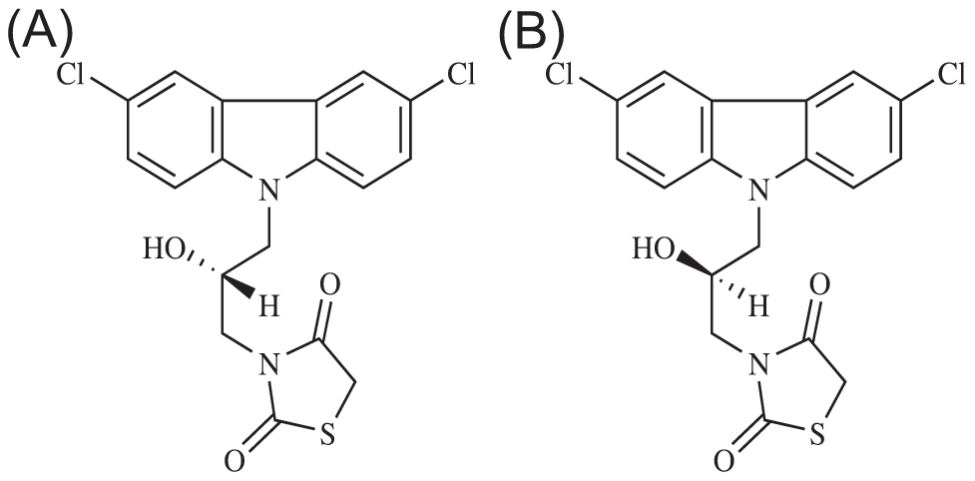
Chiral forms of the 10074-A4 ligand. **A** The S form. **B** The R form.

We conducted MD simulations with an explicit solvent model to investigate the interactions between c-Myc_370–409_ and the inhibitor 10074-A4. 10074-A4 is the only inhibitor (among seven inhibitors of c-Myc) that binds to the 375–385 sites in loop region of the bHLHZip domain of c-Myc and we wanted to see whether or not stable local structures were induced when 10074-A4 interacted with the flexible loop region. In the experimental study, 10074-A4 is a mixture of two chiral forms, the S and R forms (Figure 5). In the simulations, both chiral forms were tested. For comparison, the apo c-Myc_370–409_ was simulated with the same explicit solvent model. The accumulative simulation time for each group was 7 µs (see Methods). We calculated and compared the simulated chemical shifts with experimental chemical shifts for both implicit solvent REMD and explicit solvent simulations (Figures S7, S8, S9, S10). Reasonable agreements were found. For example, the average discrepancy between the simulated and experimental chemical shifts for Hα atoms of apo c-Myc370–409 is 0.14 and 0.16 in the MD simulations with explicit solvent model and REMD simulations, respectively (see Table S1).

The relative binding free energy of c-Myc_370–409_ with the two chiral 10074-A4 forms was analyzed from the MD trajectories using the Molecular Mechanic/Poisson-Boltzmann Surface Area (MM/PBSA) method [56]. The results of this analysis, together with the average non-bonded interactions *U*_non-bonded_ (Lennard-Jones and electrostatic potentials) between c-Myc_370–409_ and 10074-A4, are given in Table 1. We found that the interaction between c-Myc_370–409_ and the S form of 10074-A4 was much stronger than the interaction with the R form. The difference of *U*_non-bonded_ between the S and R forms (−3.7 kcal/mol) was close to the difference of Δ*H* from MM/PBSA (−3.2 kcal/mol). The difference of binding free energy between the S and R forms was −2.2 kcal/mol, resulting in a binding-affinity ratio of 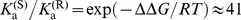 for the S and R forms. Therefore, compared with the binding of the S form to c-Myc_370–409_, the binding of the R form can be ignored. Thus, only the holo system with the S form of 10074-A4 is discussed further.

**Table 1:** Relative binding free energy and averaged non-bonded potential for 10074-A4 binding with c-Myc_370–409_ and c-Myc_410–437_.

Peptide	Ligand chirality	Δ*H*	*T*Δ*S*	Δ*G*	*U*_non-bonded_ *
c_Myc_370–409_	S	−19.9±6.5	−15.4±6.7	−4.5±13.2	−38.6±3.8
	R	−16.7±6.9	−14.3±7.8	−2.3±14.7	−34.9±4.6
c_Myc_410–437_	S	−16.6±6.9	−15.5±7.4	−1.1±14.3	−35.8±3.8
	R	−11.2±7.2	−12.8±10	1.6±17.2	−25.7±3.3

The binding free energy was calculated using the MM/PBSA method [56]. All quantities are in kcal/mol.

* *U*_non-bonded_ is averaged non-bonded potential, which is composed of Lennard-Jones and electrostatic potential.

Hammoudeh et al. [28] reported an induced circular dichroism (ICD) effect on c-Myc_370–409_ by the binding of a racemate (1∶1 mixture of the S and R forms) of 10074-A4. There were two possible reasons for the observed ICD effect [28]; either the chiral surroundings affected the absorption transition of the compound, or the enantiomer-specific effect (the different binding affinity of the S and R forms) led to the ICD effect. We have shown above that the S form of 10074-A4 bound much stronger with c-Myc_370–409_ than the R form. Therefore, we suggest that it was the enantiomer-specific effect that was responsible for the observed ICD effect. Further experiments using single chiral forms of 10074-A4 would be helpful in clarifying this observation.

**Figure 6:**
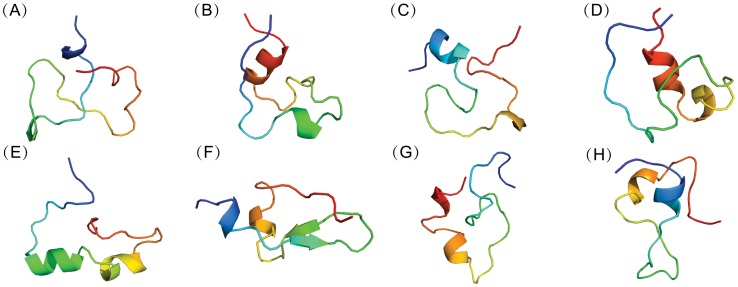
Representative conformations of apo c-Myc_370–409_ computed from explicit solvent simulations. Backbone-RMSD clustering with a cutoff of 2.0 Å of all the conformations was performed. Representative c-Myc_370–409_ structures (from blue at the N-terminal to red at the C-terminal) for the first eight clustering groups were displayed in cartoon. The fractional cluster populations are: **A** 10.5%, **B** 8.6%, **C** 7.8%, **D** 6.4%, **E** 6.1%, **F** 4.5%, **G** 3.5%, **H** 3%.

**Figure 7:**
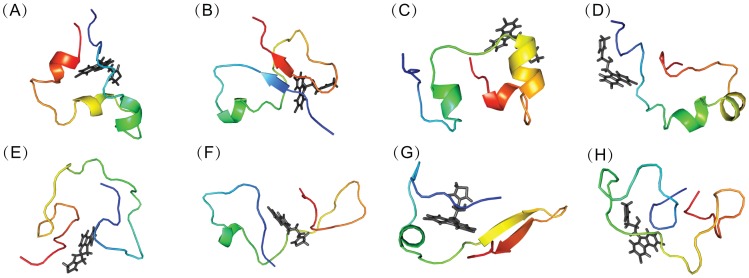
Representative conformations of holo c-Myc_370–409_/10074-A4 complex computed from explicit solvent simulations. Backbone-RMSD clustering with a cutoff of 2.0 Å of all the conformations was performed. Representative c-Myc_370–409_ structures (from blue at the N-terminal to red at the C-terminal) for the first eight clustering groups were displayed in cartoon and 10074-A4 structures were depicted as black sticks. The fractional cluster populations are: **A** 14.3%, **B** 13.9%, **C** 13.7%, **D** 10.4%, **E** 7.5%, **F** 6.9%, **G** 5.4%, **H** 5.2%.

We clustered the conformations from MD simulations with the explicit solvent model for both the apo and holo c-Myc_370–409_ peptide based on RMSD of the backbone atoms. Figure 6 and 7 showed the representative conformations for the top eight clusters of the apo and holo peptides. It is clear that both the apo and the holo peptides have a rather broad conformation distribution, which is typical of disordered proteins. Upon binding to the ligand 10074-A4, the conformational distribution became more condensed. The top eight conformation clusters of the holo peptide were more highly populated compared to that of the apo peptide, with a total of about 77% occupancy compared to 50%. Similar to the apo c-Myc_370–409_ structure, the holo c-Myc_370–409_ structure is rich in helical structures. A quantitative analysis indicated that the helix and polyproline II content was almost unaffected by the binding of 10074-A4 (Figure S11), while the sheet content was enhanced (see also in Figure 7). The electrostatic interactions (from both charged residues and hydrogen bonding) dominated the intramolecular stabilizing force for holo c-Myc_370–409_ (Figure S12).

### Interaction specificity between c-Myc_370–409_ and 10074-A4

**Figure 8:**
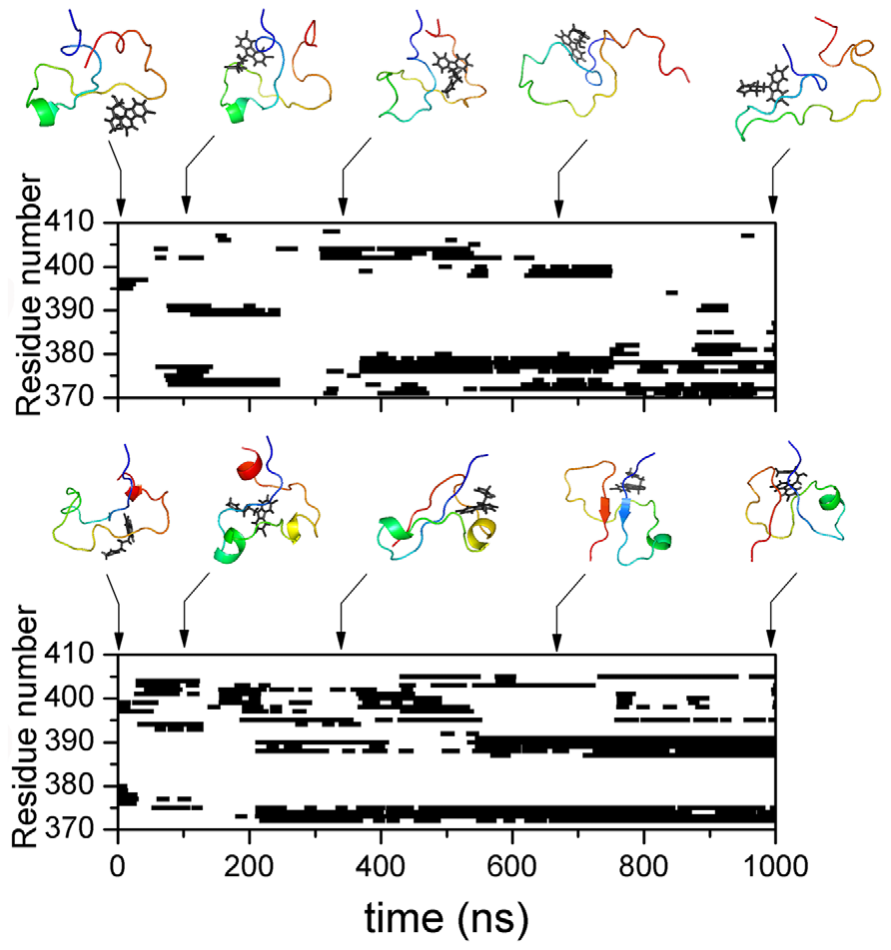
Binding sites determined as a function of time for two MD trajectories of holo c-Myc_370–409_. MD simulations with explicit solvent simulations were performed and binding sites were determined by ΔSASA. Binding residues at any time were defined by ΔSASA values larger than 10 Å^2^ and are shown in squares. Continuous binding of less than 10 ns was ignored. The results for more MD trajectories are available in Figure S13.

The residue-specific binding of c-Myc_370–409_ with 10074-A4 was tracked by calculating differences in the solvent accessible surface area (ΔSASA) between 10074-A4 and each residue of c-Myc_370–409_. The binding sites were determined as a function of time and representative conformations are shown in Figure 8. Binding of the 10074-A4 ligand was not restricted to a single site in c-Myc_370–409_, instead, it spread across almost the whole chain of c-Myc_370–409_. 10074-A4 usually binds simultaneously to two or more regions that are flanked by several residues. The binding was highly dynamic and could switch between different modes within a trajectory.

**Figure 9:**
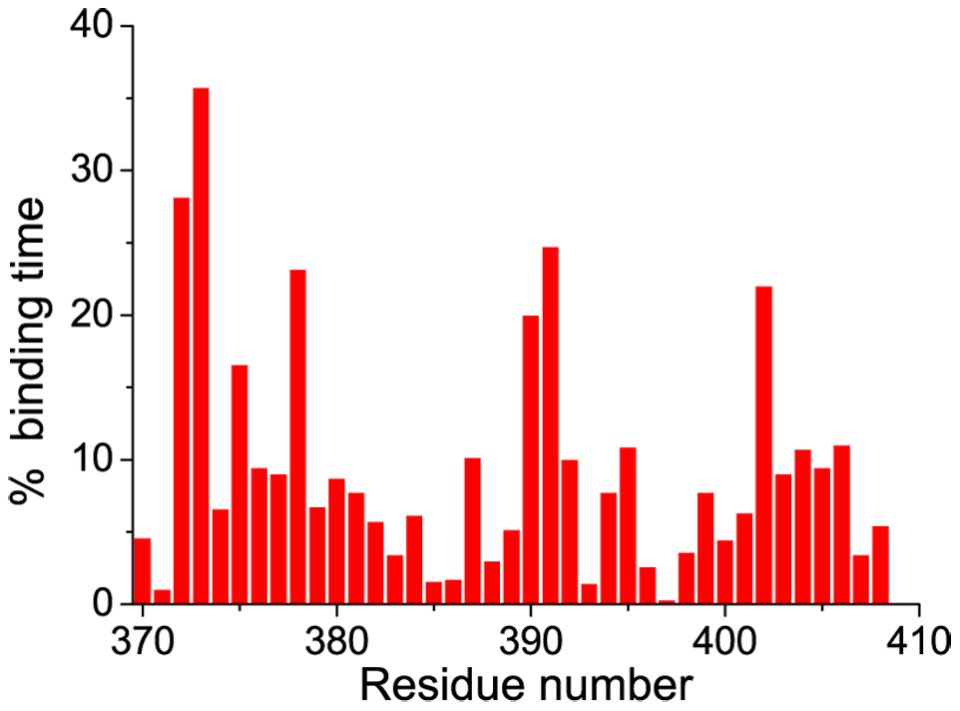
Binding specificity of 10074-A4 with c-Myc_370–409_. The binding-time percentage was computed for each residue by counting the frames with ΔSASA larger than 10 Å^2^. Continuous binding of less than 10 ns was ignored.

**Figure 10:**
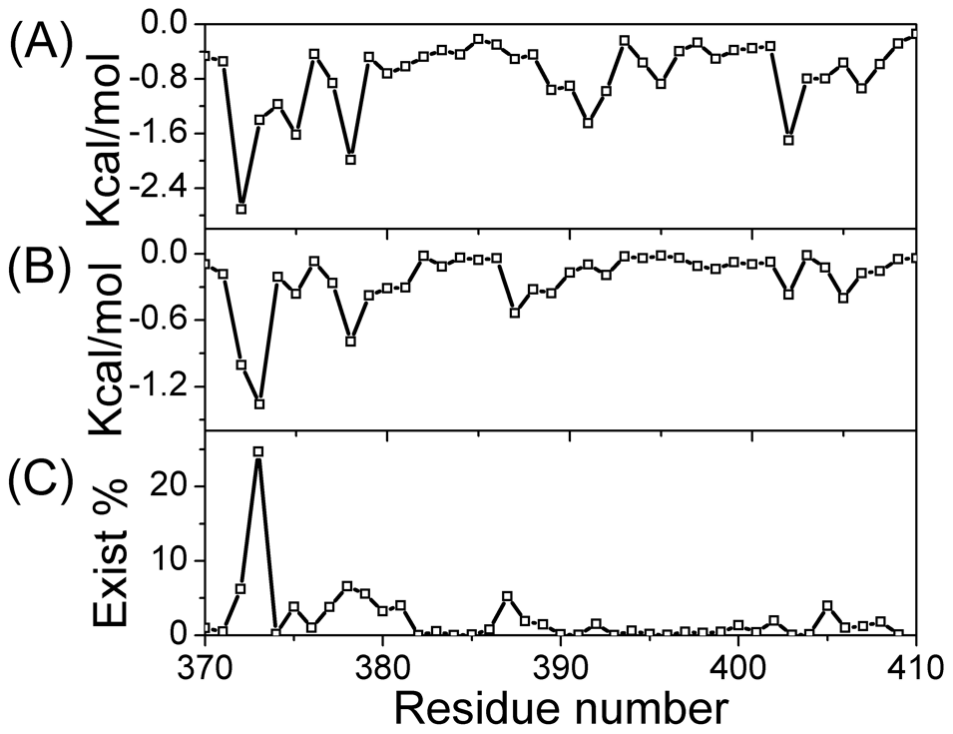
Interactions between 10074-A4 and the c-Myc_370–409_ peptide. **A** Lennard-Jones potential. **B** Electrostatic potential. **C** Time percentage of hydrogen bonds.

The time percentage of binding for each residue was calculated and is shown in Figure 9. Three binding sites were detected, which included site I (residues 372 to 384), site II (387 to 395), and site III (398 to 408). Site I was near the N-terminal and showed stronger potency than that of the other two sites. This result was supported by the intermolecular interaction analysis (Figure 10), which showed that both the electrostatic and Lennard-Jones interactions for site I were much stronger than those of the other two sites. In fact, in the latter cases, hydrogen bonds hardly formed and the electrostatic interactions were weak. Site I was similar to the experimentally determined binding site of 10074-A4 on c-Myc at residues 374–385 [28]. Binding at all the other sites generated in our simulations was much weaker, which would make them difficult to be observed experimentally. The low residue interaction specificity that we observed in the simulations is consistent with a recent simulation on an 11 residue peptide of c-Myc_402–412_ that suggested that ligand binding was driven by weak and nonspecific interactions [43]. The mass spectrometry experiment on c-Myc reported by Harvey et al. [32] also supported this conclusion.

### Negative control study with c-Myc_410–437_

**Figure 11:**
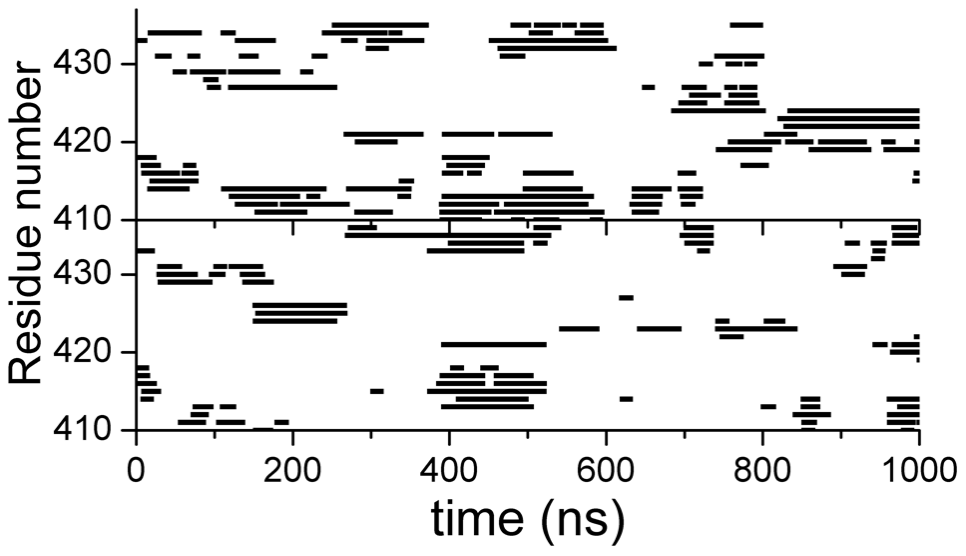
Binding of c-Myc_410–437_ with the S (upper) and R (lower) forms of 10074-A4.

To further investigate the inherent specificity features of IDPs, we conducted a negative control study in which we chose another segment of c-Myc (residues 410–437) that does not bind with 10074-A4 [28]. The simulated binding between c-Myc_410–437_ and 10074-A4 is shown in Figure 11. Unexpectedly, c-Myc_410–437_ “bound” with 10074-A4 in most simulation durations. Comparing with the binding of 10074-A4 with c-Myc_370–409_, its binding with c-Myc_410–437_ was less lasting and switched more frequently among different modes. The longest continuous binding time at one binding region within a trajectory is about 800 ns for c-Myc_370–409_ (see lower part of Figure 8), while it is about 200 ns for c-Myc_410–437_ (Figure 11).

The observed “binding” in the c-Myc_410–437_ negative control was different from what is found in negative controls for conventional ordered proteins where binding is usually not observed. To clarify the nature of this unexpected finding, we calculated the relative binding free energy using the MM/PBSA method and the results are provided in Table 1. We found that the binding of 10074-A4 with c-Myc_410–437_ was much weaker than with c-Myc_370–409_; the difference in binding free energy was about 3.4 kcal/mol. Therefore, the binding in c-Myc_410–437_ could not compete with that in c-Myc_370–409_. Although 10074-A4 scattered around the c-Myc_370–409_ and c-Myc_410–437_ peptides (Figures 8 and 11), its interaction with c-Myc_370–409_ was stronger and more selective than with c-Myc_410–437_. The sites at which 10074-A4 “bound” with the c-Myc_410–437_ peptide were much more disperse than the sites at which it bound with c-Myc_370–409_. Therefore, though the binding of 10074-A4 and c-Myc_370–409_ was not strong (the experimentally determined dissociate constant was 21±2 µM), it showed selectivity and thus specificity.

## Discussion

**Figure 12:**
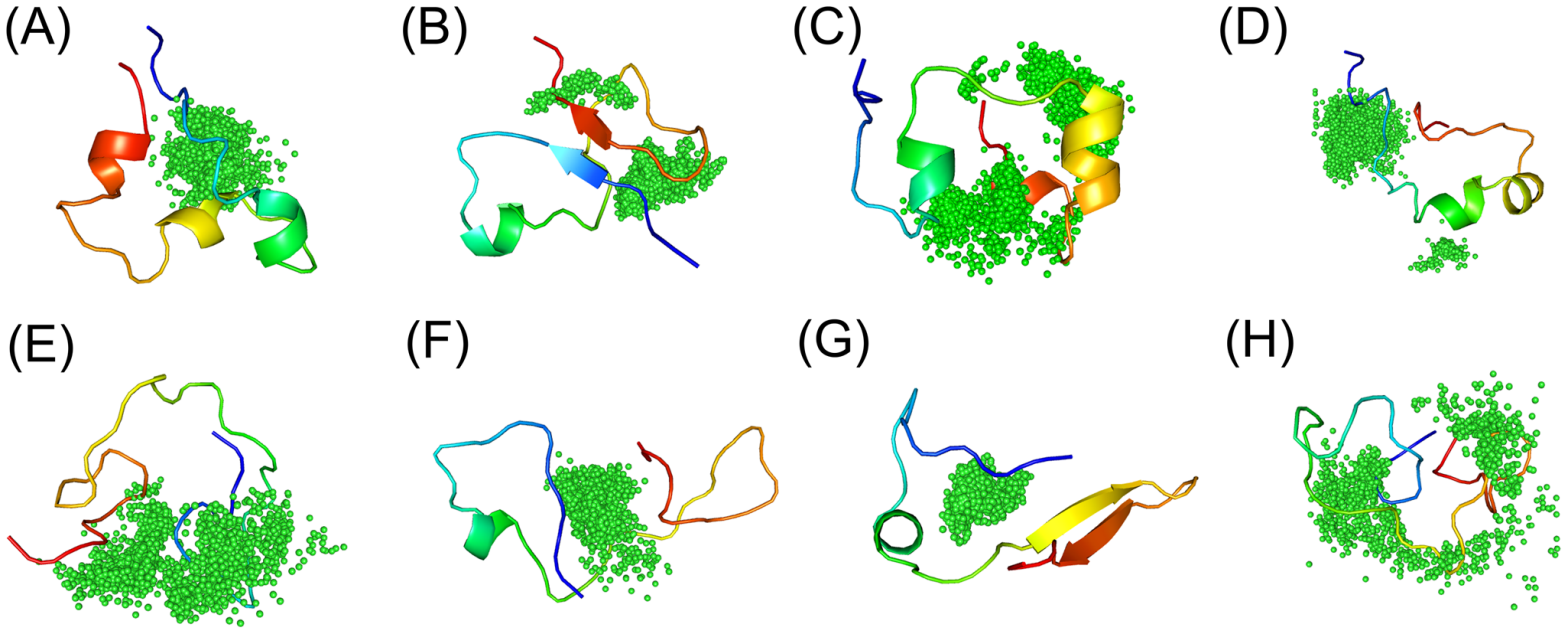
Illustration of the ligand clouds concept. Holo conformations from the simulations were clustered and representative c-Myc_370–409_ structures of each clustering group were displayed in the same way as Figure 7. Ligand 10074-A4 structures from each group were depicted as green dots at the centers of mass.

The specificities of IDPs in molecular recognition are complicated [57]. Our simulation results showed that the specificity of c-Myc in binding the small-molecule ligand 10074-A4 was not high. C-Myc is a typical example of IDPs. It is sticky and binds the ligands at different regions with different interaction strengths. Because of the lack of coupled folding and binding, after binding, c-Myc is still in an ensemble with diverse conformations and the distinct conformations are all capable of binding the ligand. Furthermore, for a given c-Myc structure, the binding of ligand occurred at disperse sites (Figure 12). We named this phenomenon ligand clouds. Ligand clouds are remarkably different from the type of binding that is found in ordered proteins where a dominant binding structure is formed. We expect that ligand clouds may be a general feature for IDPs binding with small-molecule ligands. For IDPs binding with macromolecule partners, it was reported that some IDPs remain disordered in the holo state [57]; for example, β-catenin/Tcf4, β-catenin/APC peptide, β-catenin/APC phosphorylated, Vif/EloB/EloC, and ERRγLBD/PGC-1α. These IDP complexes assume dynamic structures upon binding, suggesting that IDPs may interact with their partners in a similar manner to the ligand clouds. The ligand clouds concept supports the idea that there is no definite binding mode in the interactions between IDPs and small-molecule inhibitor [43]. It suggests that the interactions could be described as protein clouds interacting with ligand clouds.

The ligand cloud concept describes a scenario for the interactions between IDPs and small-molecule ligands and may provide a basis for drug design targeting IDPs. A straightforward strategy for rational drug design on IDPs is to extract metastable structures from simulations and then to conduct a virtual screen on them to identify potential inhibitors. A similar strategy was applied successfully in designing an inhibitor for Aβ fibrillation [58]. However, the ligand clouds concept for small molecules binding with IDPs implies that different strategies from those used for ordered proteins should be developed for better rational drug design on IDPs. For example, because ligand binding on IDPs occurs in disperse locations and in different orientations, multimode interactions should be considered in the scoring functions instead of the single-mode interaction that is commonly used for other proteins. Therefore, schemes that can consider binding energy landscapes [59] might be expected to perform better when designing small molecule ligands for IDPs. On the other hand, in contrast to the conventional ordered proteins that are in either “binding” or “non-binding” states with small molecules, IDPs are “sticky” and would be either in “strong binding” or “weak binding” with small molecules. So more cares should be paid to the problem of specificity in drug design targeting IDPs.

For conventional ordered proteins, the binding conformation is unique which could be selected from pre-existing conformations (the conformational selection mechanism) or be induced (the induced fit mechanism) by particular ligands. The scenario of ligand clouds around protein clouds for IDPs indicates that multiple protein conformations are selected and/or induced by the binding of a ligand on IDPs. This may extend the conformational selection-induced fit continuum in a new dimension.

### Conclusion

In conclusion, we conducted extensive simulations to explore the conformational ensemble of c-Myc_370–409_ and its complex with a small-molecule inhibitor 10074-A4. The conformational space was found to be rather dispersed. In contrast to conventional structured proteins, the conformations of c-Myc_370–409_ were mainly stabilized by charge interactions and hydrogen bonds. Upon binding to 10074-A4, c-Myc_370–409_ remained disordered. The 10074-A4 ligand bound at different sites throughout the c-Myc_370–409_ chain with different strength. Accordingly, a ligand cloud concept was proposed, that is, the interactions between small molecule ligands and IDPs were like ligand clouds around protein clouds. The different binding probabilities between the protein clouds and ligand clouds indicated that the ligand could be selective and thus specific. Though the specificity of the binding was not high, the binding of ligand and non-ligand to the target IDP could be clearly distinguished.

## Methods

### NMR structures of c-Myc_370–409_ and its complex with 10074-A4

Hammoudeh et al. [28] measured chemical shifts and several NOE signals of c-Myc_370–409_ and predicted dihedral angle distributions and atomic contacts. To build the c-Myc_370–409_ peptide, we first built a completely extended conformation with the following sequence: ^370^ LKRSFFALRDQIPELENNEKAPKVVILKKATAYILSVQAE ^409^ (Accession number: P01106). We then built the initial structures from the reported dihedral angles [28] using PyMOL [60]. The apo and holo structures for c-Myc_370–409_ were refined further using the GROMACS 4.5.4 software package [61] and the AMBER99SB force field, with the NMR data [28] as the dihedral angle and distance restraints in the simulation. Each initial structure was minimized in vacuum. Then, it was solvated, minimized, and equilibrated as described below. The time step was set to 0.5 fs. Finally, a 5 ns production simulation was performed and the final structure was adopted as the refined structure.

### REMD simulations with implicit solvent model

The conformations of the c-Myc_370–409_ peptide were sampled by REMD simulations with a Generalized Born/Surface Area (GB/SA) implicit solvent model. The AMBER molecular simulations package was used with AMBER99SB force fields [62]. A total of 30 replicas were adopted with temperatures ranging between 284.6 K and 608.8 K. All adjacent replicas attempted to exchange temperature every 10 ps with the average exchange rate between 35% and 40%. To produce the 30 starting conformations for an REMD simulation, an initial structure (described below) was minimized using steepest descent for 500 steps and then switched to conjugate gradient for another 500 steps. The minimized conformation was then heated to the defined temperature over a time of 200 ps for each replica. The obtained conformations were adopted as starting conformations in the REMD simulations, which were run with a time step of 2 fs. Replica temperature was controlled with a coupling time constant of 2 ps. Bonds involving hydrogen atoms were constrained with SHAKE. Chirality restraints on the backbone were employed to prevent non-physical chiralities. Ionic strength was set to 0.2 M. The cutoff for non-bonded interactions and for the GB pairwise summations involved in calculating Born radii was 999 Å to consider all probable interactions entirely. Snapshots from each trajectory were stored every 10 ps.

We conducted four groups of REMD simulations with different initial structures: (a) the extended structure of the peptide; (b) apo NMR refined structure; (c) the structure after a 80-ns MD simulation at 300 K starting from the extended conformation; and (d) the most occupied representative conformation generated previously from the REMD simulations of the extended structure in (a). The simulation time for the four groups of REMDs was 150 ns, 270 ns, 210 ns and 520 ns, respectively. The total simulation time was 34.5 µs (1.15 µs per replica).

The trajectories of 292.2 K, 300 K and 308 K were used in the further analyses except that only the trajectory of 300 K was used in the chemical shifts calculations.

### MD simulations with explicit solvent model

To investigate the interactions between c-Myc_370–409_ and 10074-A4, MD simulations for the complex structure were carried out with an explicit solvent model [63]. The apo c-Myc_370–409_ was also simulated with the same explicit solvent model for comparison. Three groups of simulations were performed, one for the apo and two for the holo (with the two chiral 10074-A4 forms (see Figure 5)). Each group contained seven trajectories of 1 µs, therefore, the total simulation time was 21 µs. One of the seven initial structures was the NMR refined structures (apo and holo); the other six initial structures were adopted from representative conformations generated previously in the 150-ns REMD simulations (for the holo structures, the 10074-A4 isomers were docked using the AutoDock 4.2 program [64]).

MD simulations with the explicit solvent model were performed with the GROMACS 4.5.4 software package [61] and AMBER99SB force field under particle mesh Ewald periodic boundary conditions. The TIP4P-EW water model [63] was used with AMBER99SB force field because of its previously reported good performance in other simulations of IDPs [36], [47], [65]. In the holo simulations, the small molecule 10074-A4 ligand involved was parameterized using a general amber force field (gaff) with ACPYPE software [66]. An AM1-BCC charge model [67] was used to assign charges to the ligand.

Each initial structure was immersed in an explicit TIP4P-EW truncated octahedral water box. The dimensions of the box, defined as the distance between the farthest atoms of the peptide and the edge of the box, was set to 10 Å. The system was neutralized by adding ions, and extra NaCl was added to represent a solution with an ionic strength of 0.15 M. The system was minimized using the steepest descent minimization approach. After the minimization, the system was equilibrated in the NVT ensemble with all-heavy atom restrained with a force constant of 239 kcal/mol. The temperature was maintained at 300 K using a V-rescale thermostat with a coupling constant of 0.1 ps. Further equilibration was carried on in the NPT ensemble without strains, and where the pressure was maintained at 1 atmosphere using a Parrinello-Rahamn barostat with the coupling constant set to 2.0 ps. Both equilibrations were performed for 200 ps with a time step of 1 fs. For the production run, the thermostat and barostat settings were the same as for the NPT run. To enable 2 fs time steps, bonds involving hydrogen atoms were constrained to equilibration length using the LINCS algorithm [68]. A real-space cutoff of 10 Å was used for the electrostatic and Lennard-Jones forces. Snapshots from each trajectory were stored every 20 ps.

To further investigate the inherent specificity features of IDPs, we conducted a negative control study using the c-Myc_410–437_ truncated peptide (^410^EQKLISEEDLLRKRREQLKHKLEQLRNS^437^), which did not bind to 10074-A4. The extended structure of the peptide was used as the initial structure in an 80 ns implicit solvent MD simulation and the final structure that was generated was applied in all-atom explicit simulations. Two groups of simulations were performed for each of the two chiral 10074-A4 isomers. Each group contained one trajectory of 1 µs; the other parameters were the same as the parameters used for the holo c-Myc_370–409_ simulations described above.

### Analysis of the simulations

All the simulations were analyzed using the GROMACS utilities [61] with either PyMol [60] or in-house scripts. ΔSASA was used in determinations of the binding sites. Upon small molecule binding, for each residue in the peptide there would be a clear decrease of SASA related to the difference between the SASA of the bound and unbound states. Backbone RMSD clustering of peptide conformations was performed to identify distinct structural clusters and to estimate their populations. The relative binding free energy was calculated every 200 ps using MM/PBSA [56] methods.

## Supporting Information

Figure S1 **Comparisons of the computed and experimental chemical shifts for apo c-Myc_370–409_.** The computed values using SHIFTX (red circles), CamShift (blue square) and SPARTA+ (green squares) are from the REMD simulations and the experimental values are from Hammoudeh et al. [28] (black triangle). Note that the experimental values for some residues were not available. Chemical shifts are for the atoms: **A** H_α_, **B** H_N_, **C** C_α_, **D** C_β_.
(TIF)

Figure S2 **Comparisons of the back-calculated chemical shifts for NMR-refined apo c-Myc_370–409_ structure and experimental values.** The computed values for apo c-Myc_370–409_ were obtained using SHIFTS (red circles) and SHIFTX (blue triangles). The experimental values for apo c-Myc_370–409_ are from Hammoudeh et al. [28] (green squares). Note that the experimental values for some residues were not available.
(TIF)

Figure S3 **Distribution of chemical shifts for apo c-Myc_370–409_ determined from REMD simulations.** **A** Chemical shifts for the H_N_ atoms. **B** Chemical shifts for the C_α_ atoms. **C** Chemical shifts for the C_β_ atoms. Experimental values are indicated by red arrows for comparison.
(TIF)

Figure S4 **Ramachadran plots for the apo c-Myc_370–409_ dihedral angles computed from implicit solvent REMD simulations.** The backbone dihedral angle values estimated from the experimental structure are indicated by blue crosses for comparison.
(TIF)

Figure S5
**Dimension and helix content distributions of apo c-Myc_370–409_.** **A** Distribution of radius of gyration for conformations obtained from REMD simulations. The radius of gyration of native state and denatured state (random coils) were computed using empirical formulas 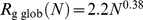 and 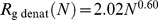 [38], where N is the number of residues, and are indicated by arrows in the figure. **B** Distribution of helix content of conformations from REMD simulations.(TIF)

Figure S6 **Residue-residue interactions in apo c-Myc_370–409_ computed from REMD simulations.** **A** Lennard-Jones potential (in kcal/mol). **B** Contact map (in contact probability). **C** Electrostatic potential (in kcal/mol). **D** Time percentage of hydrogen bonds. An *i*-*j* residue pair was defined as in contact when an atom in the *i*th residue and an atom in the *j*th residue were closer than 4.0 Å and *j*>*i*+2.
(TIF)

Figure S7 **Comparisons of chemical shifts for apo c-Myc_370–409_ computed from explicit solvent simulations (red circles) and the experimental values of Hammoudeh et al.** [**28**] **(blue squares).** Note that the experimental values for some residues were not available.
(TIF)

Figure S8 **Distribution of H_α_ chemical shifts for apo c-Myc_370–409_ determined from explicit solvent simulations.** Experimental values are indicated by red arrows for comparison.
(TIF)

Figure S9 **Comparisons of chemical shifts for holo c-Myc_370–409_ computed from holo explicit solvent simulations (red circles) and the experimental values of Hammoudeh et al.** [**28**] **(blue squares).** Note that the experimental values for some residues were not available.
(TIF)

Figure S10 **Distribution of H_α_ chemical shifts for holo c-Myc_370–409_ determined from explicit solvent simulations.** Experimental values are indicated by red arrows for comparison.
(TIF)

Figure S11 **Secondary structure content of apo (black) and holo (red) c-Myc_370–409_ computed from explicit solvent simulations.** The helix and sheet content was computed using the DSSP method [52]; the polyproline II content was computed with the PROSS software [53].
(TIF)

Figure S12 **Residue-residue interactions in apo (upper) and holo (lower) c-Myc_370–409_ computed from explicit solvent simulations.** **A** and **E** Lennard-Jones potential (in Kcal/mol). **B** and **F** Contact map (in contact probability). **C** and **G** Electrostatic potential (in Kcal/mol). **D** and **H** Time percentage of hydrogen bonds. An *i*-*j* residue pair was defined as in contact when an atom in the *i*th residue and an atom in the *j*th residue were closer than 4 Å and *j*>*i*+2.
(TIF)

Figure S13 **Binding sites of holo c-Myc_370–409_ determined by ΔSASA as a function of time for five MD trajectories with explicit solvent simulations.** Binding residues were defined by ΔSASA larger than 10 Å^2^ and are shown in squares. Continuous binding of less than 10 ns was ignored.
(TIF)

Table S1 **Average discrepancy between simulated and experimental chemical shifts for H_α_ atoms of apo c-Myc_370–409_ calculated using SHIFTS.**
(TIF)
