# Supplementary figures and images for: Ligand Clouds around Protein Clouds: A Scenario of Ligand Binding with Intrinsically Disordered Proteins
Source: PLoS Comput Biol. 2013 Oct 3;9(10):e1003249. doi: 10.1371/journal.pcbi.1003249 (PMC3789766; doi:10.1371/journal.pcbi.1003249)

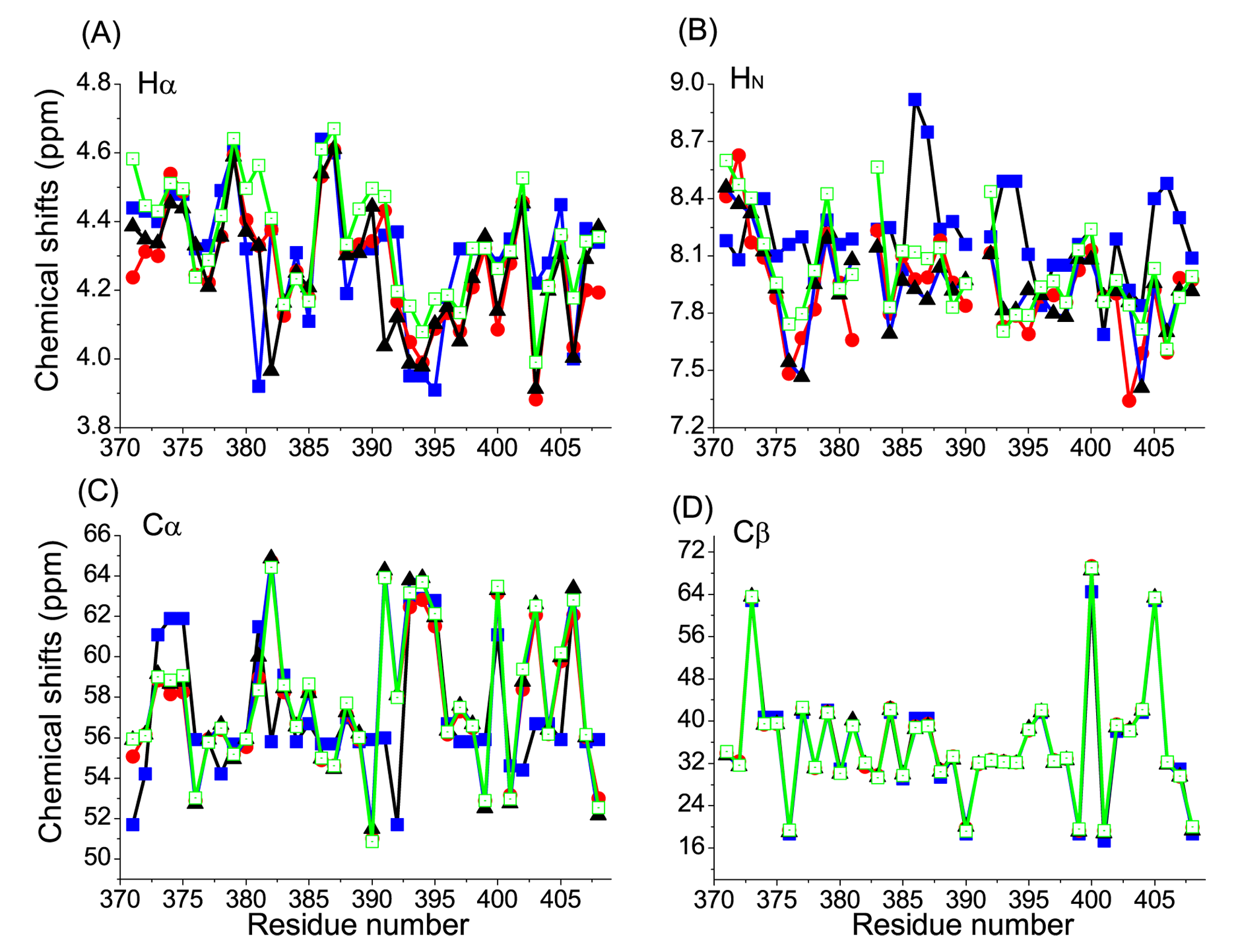

Supplement: Figure S1 — Comparisons of the computed and experimental chemical shifts for apo c-Myc370–409. The computed values using SHIFTX (red circles), CamShift (blue square) and SPARTA+ (green squares) are from the REMD simulations and the experimental values are from Hammoudeh et al. [28] (black triangle). Note that the experimental values for some residues were not available. Chemical shifts are for the atoms: A Hα, B HN, C Cα, D Cβ. (TIF) [file pcbi.1003249.s001.tif]

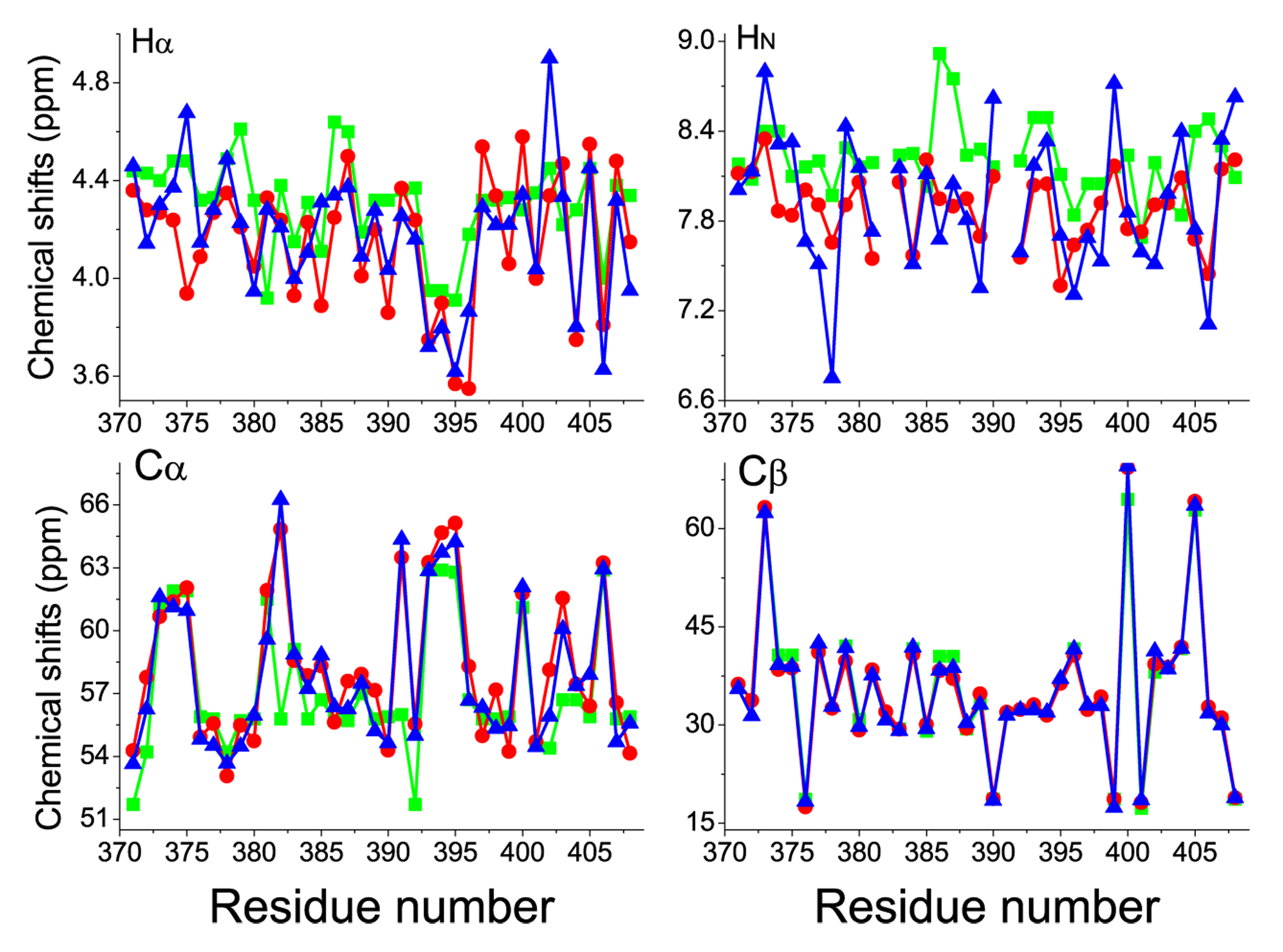

Supplement: Figure S2 — Comparisons of the back-calculated chemical shifts for NMR-refined apo c-Myc370–409 structure and experimental values. The computed values for apo c-Myc370–409 were obtained using SHIFTS (red circles) and SHIFTX (blue triangles). The experimental values for apo c-Myc370–409 are from Hammoudeh et al. [28] (green squares). Note that the experimental values for some residues were not available. (TIF) [file pcbi.1003249.s002.tif]

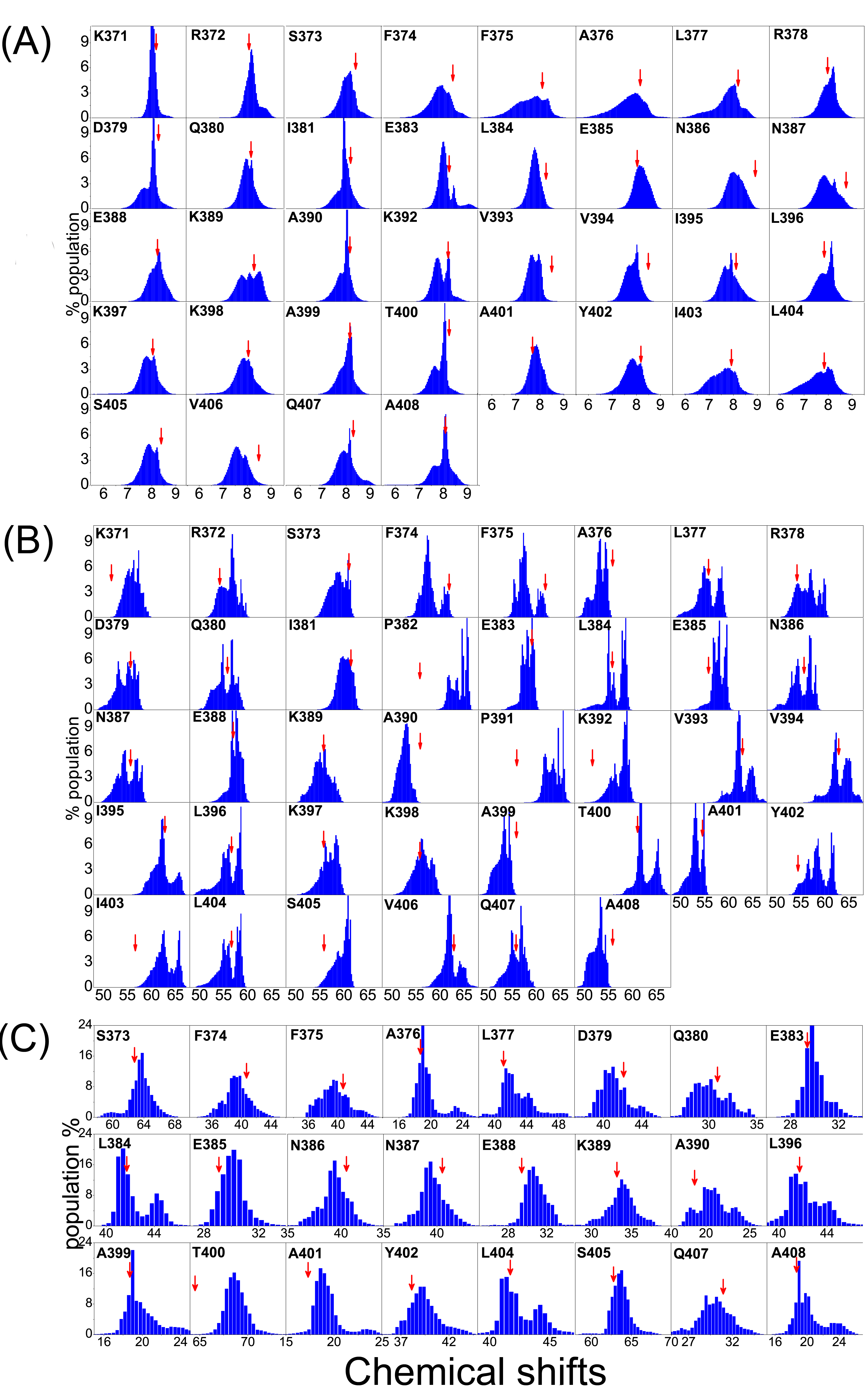

Supplement: Figure S3 — Distribution of chemical shifts for apo c-Myc370–409 determined from REMD simulations. A Chemical shifts for the HN atoms. B Chemical shifts for the Cα atoms. C Chemical shifts for the Cβ atoms. Experimental values are indicated by red arrows for comparison. (TIF) [file pcbi.1003249.s003.tif]

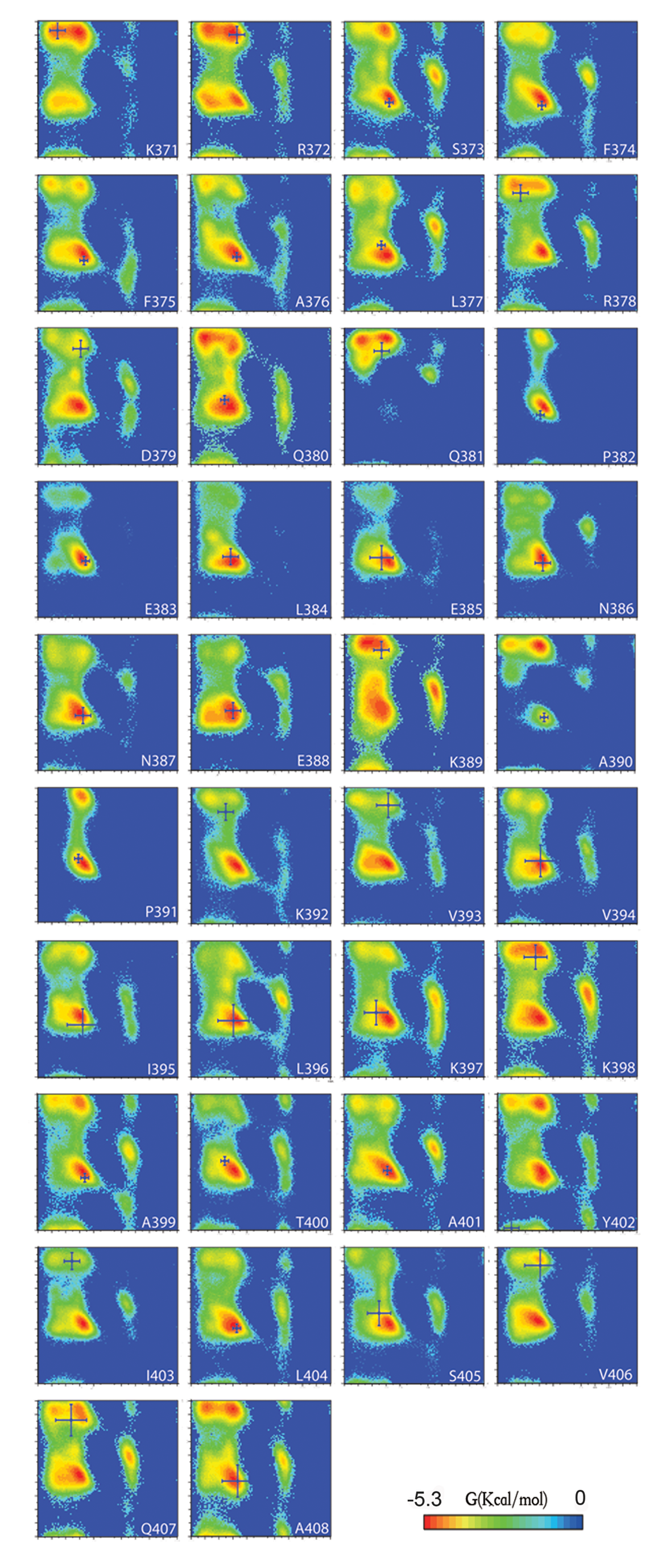

Supplement: Figure S4 — Ramachadran plots for the apo c-Myc370–409 dihedral angles computed from implicit solvent REMD simulations. The backbone dihedral angle values estimated from the experimental structure are indicated by blue crosses for comparison. (TIF) [file pcbi.1003249.s004.tif]

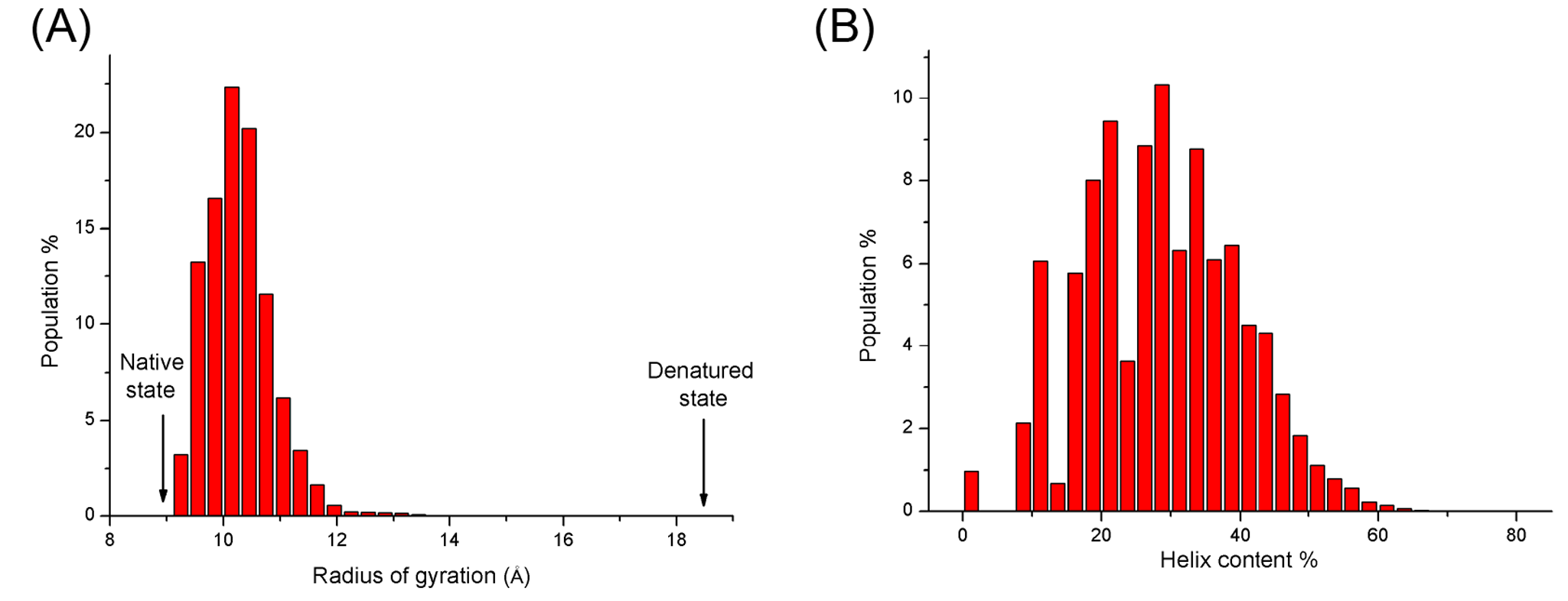

Supplement: Figure S5 — Dimension and helix content distributions of apo c-Myc370–409. A Distribution of radius of gyration for conformations obtained from REMD simulations. The radius of gyration of native state and denatured state (random coils) were computed using empirical formulas and [38], where N is the number of residues, and are indicated by arrows in the figure. B Distribution of helix content of conformations from REMD simulations. (TIF) [file pcbi.1003249.s005.tif]

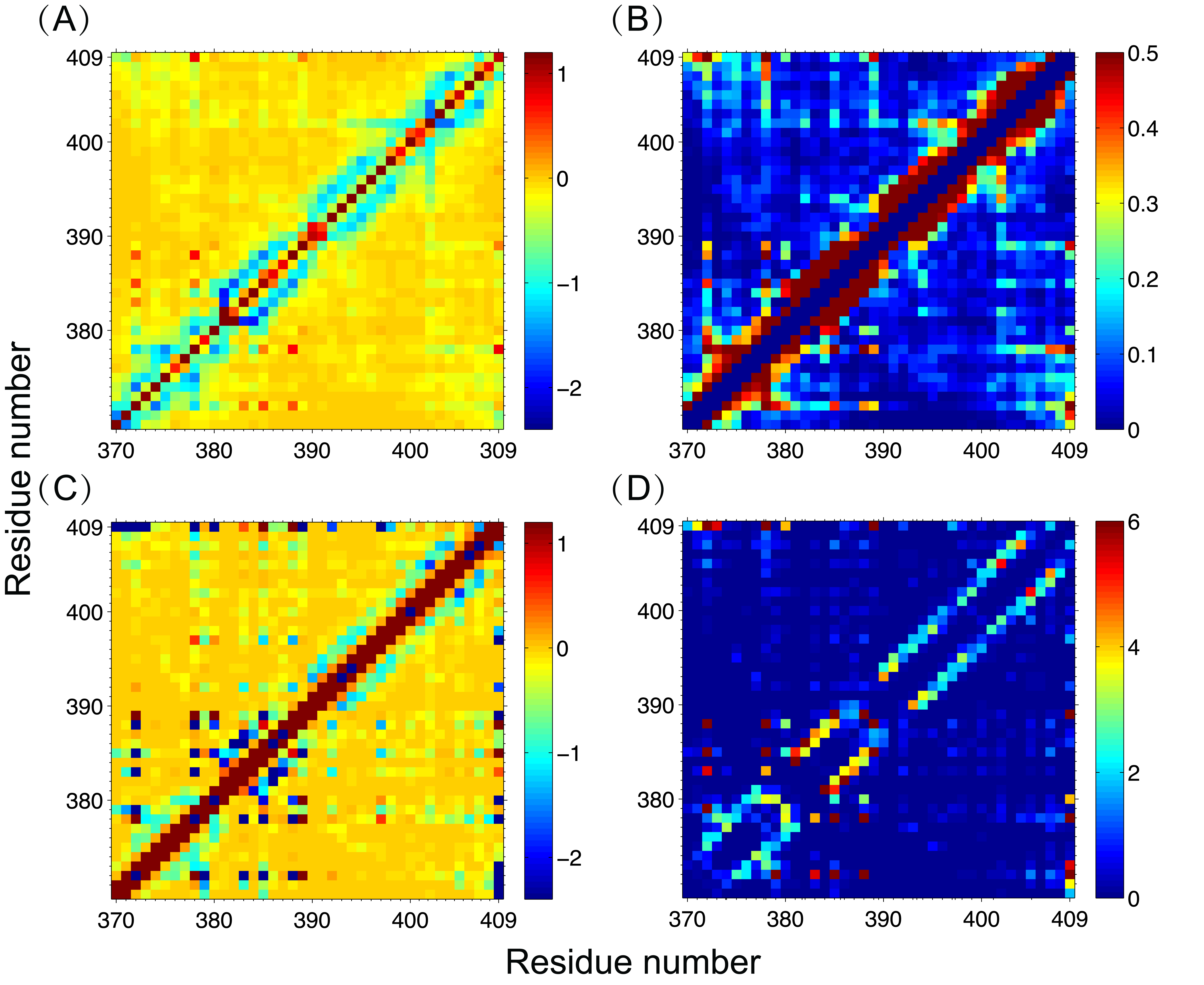

Supplement: Figure S6 — Residue-residue interactions in apo c-Myc370–409 computed from REMD simulations. A Lennard-Jones potential (in kcal/mol). B Contact map (in contact probability). C Electrostatic potential (in kcal/mol). D Time percentage of hydrogen bonds. An i-j residue pair was defined as in contact when an atom in the ith residue and an atom in the jth residue were closer than 4.0 Å and j>i+2. (TIF) [file pcbi.1003249.s006.tif]

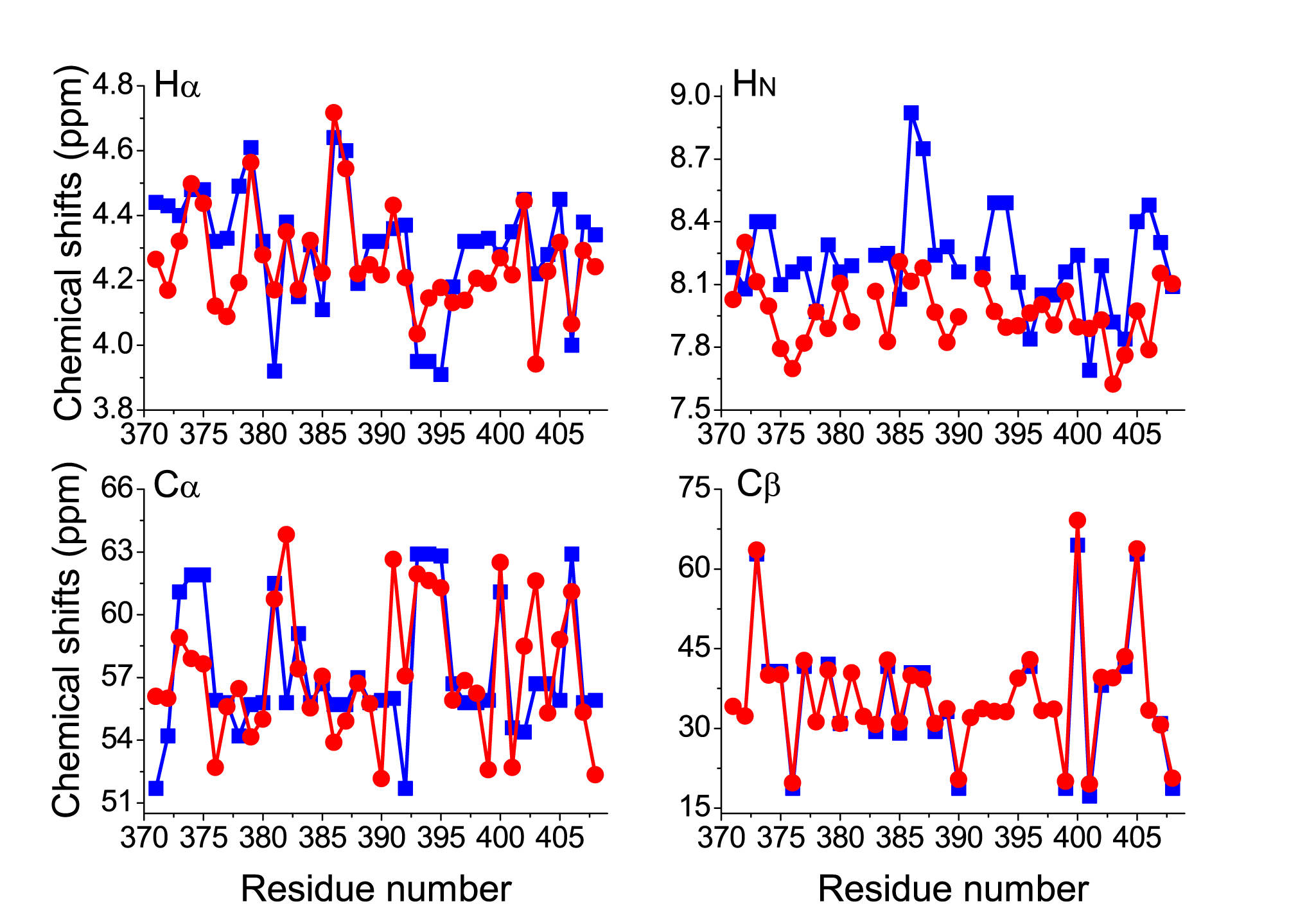

Supplement: Figure S7 — Comparisons of chemical shifts for apo c-Myc370–409 computed from explicit solvent simulations (red circles) and the experimental values of Hammoudeh et al. [28] (blue squares). Note that the experimental values for some residues were not available. (TIF) [file pcbi.1003249.s007.tif]

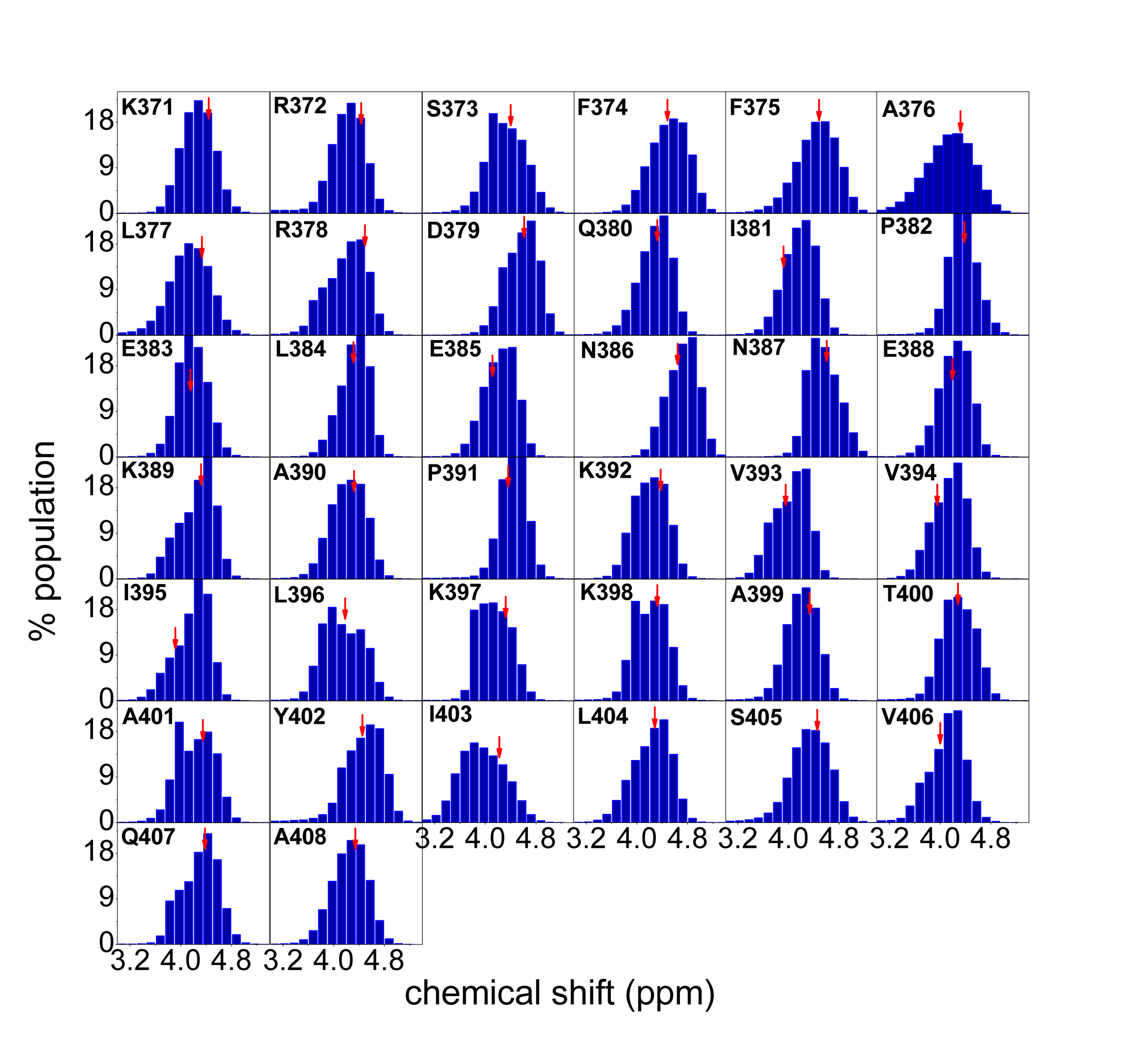

Supplement: Figure S8 — Distribution of Hα chemical shifts for apo c-Myc370–409 determined from explicit solvent simulations. Experimental values are indicated by red arrows for comparison. (TIF) [file pcbi.1003249.s008.tif]

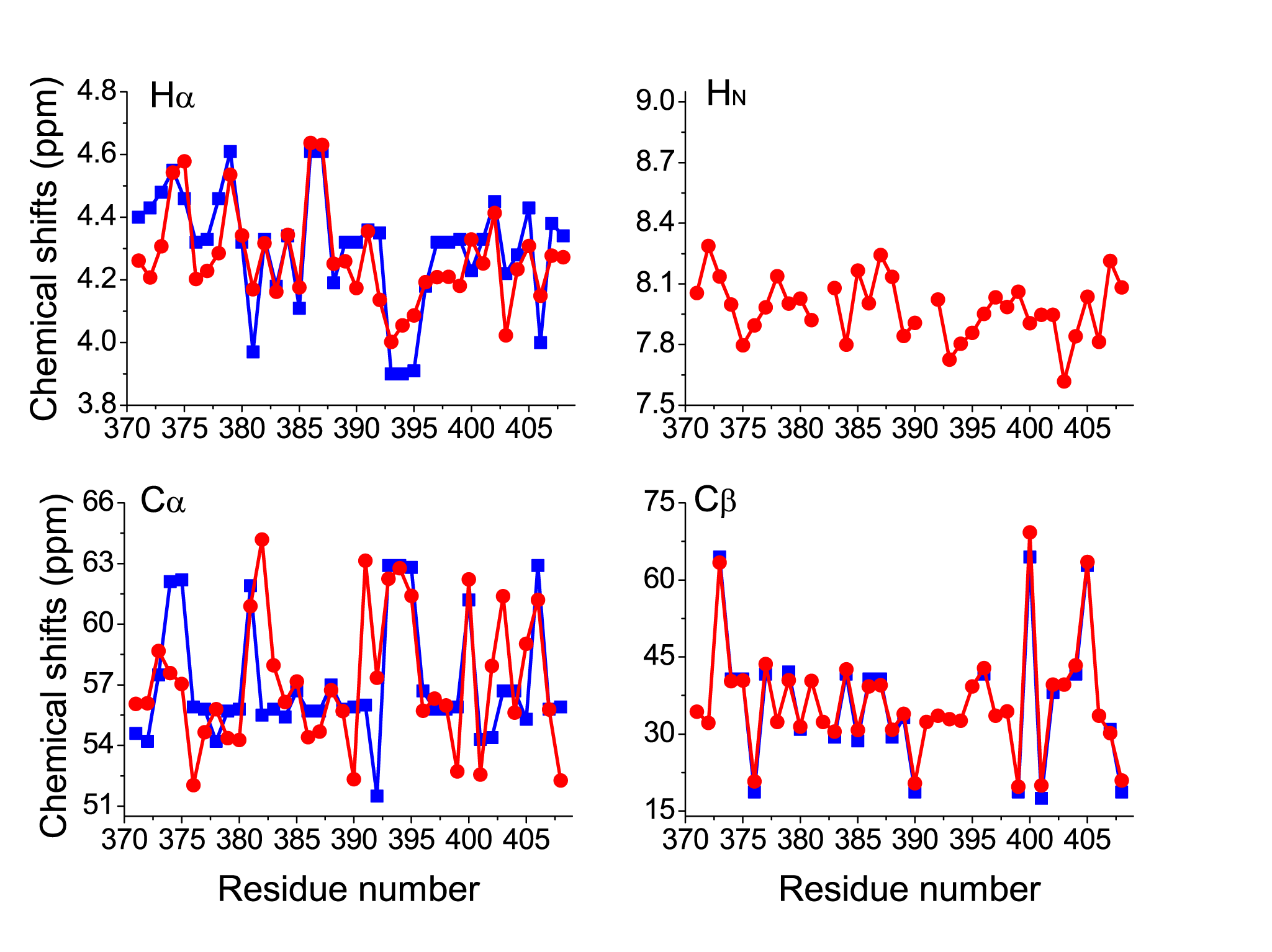

Supplement: Figure S9 — Comparisons of chemical shifts for holo c-Myc370–409 computed from holo explicit solvent simulations (red circles) and the experimental values of Hammoudeh et al. [28] (blue squares). Note that the experimental values for some residues were not available. (TIF) [file pcbi.1003249.s009.tif]

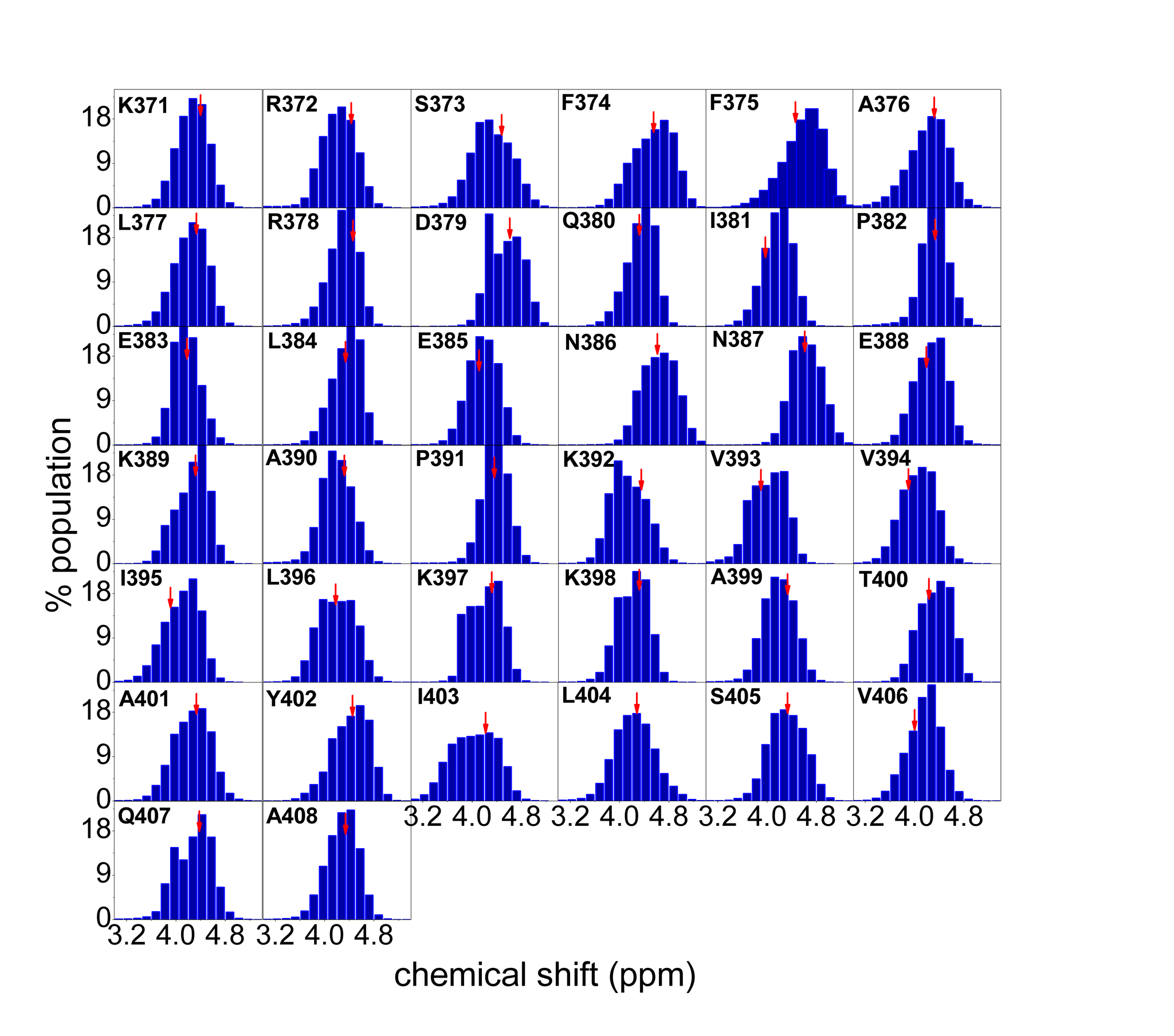

Supplement: Figure S10 — Distribution of Hα chemical shifts for holo c-Myc370–409 determined from explicit solvent simulations. Experimental values are indicated by red arrows for comparison. (TIF) [file pcbi.1003249.s010.tif]

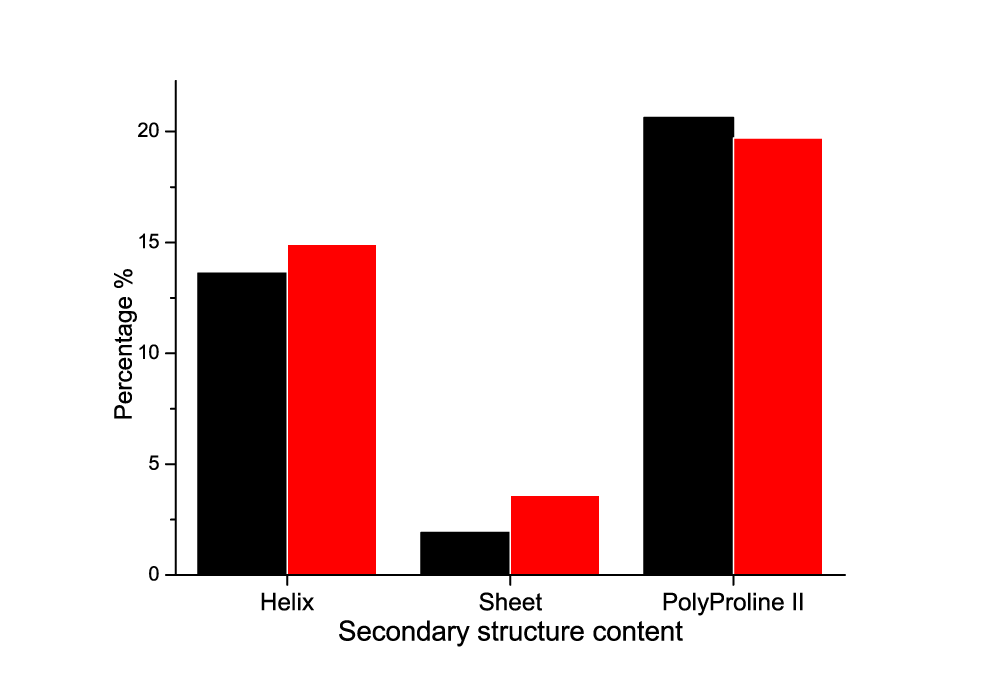

Supplement: Figure S11 — Secondary structure content of apo (black) and holo (red) c-Myc370–409 computed from explicit solvent simulations. The helix and sheet content was computed using the DSSP method [52]; the polyproline II content was computed with the PROSS software [53]. (TIF) [file pcbi.1003249.s011.tif]

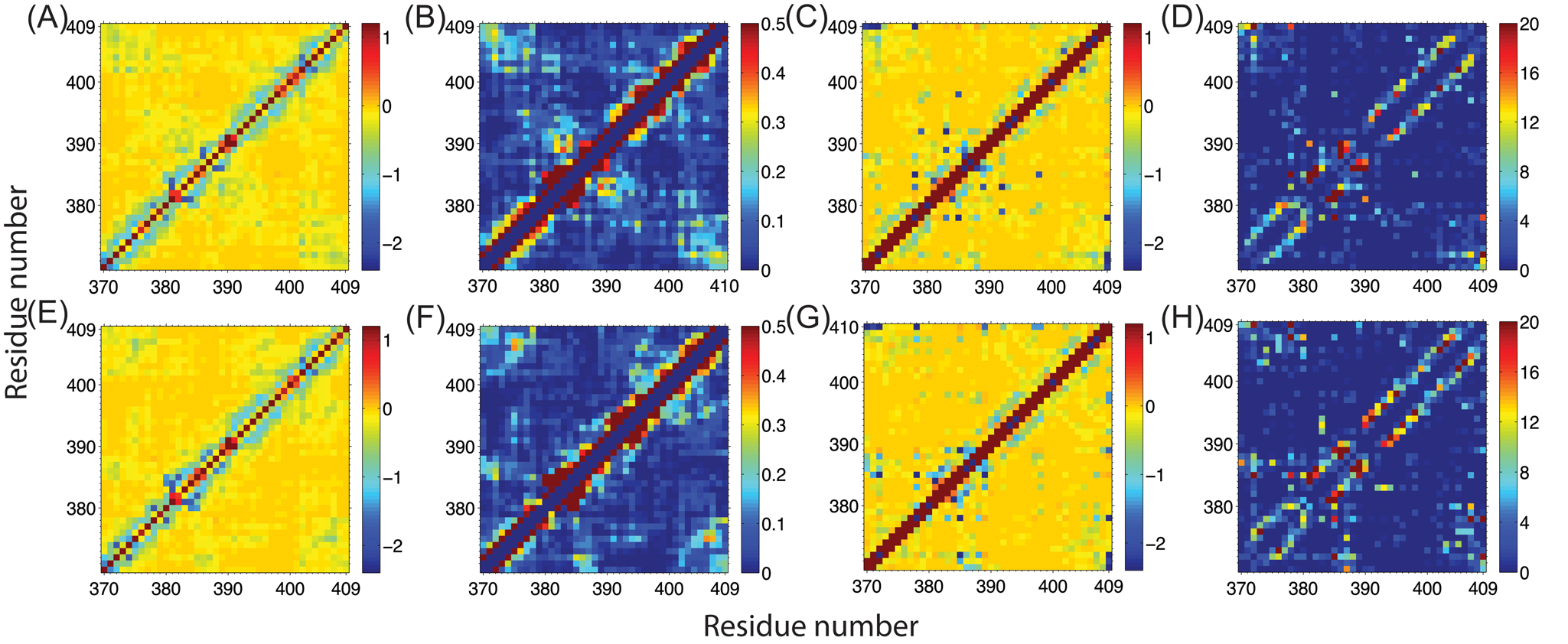

Supplement: Figure S12 — Residue-residue interactions in apo (upper) and holo (lower) c-Myc370–409 computed from explicit solvent simulations. A and E Lennard-Jones potential (in Kcal/mol). B and F Contact map (in contact probability). C and G Electrostatic potential (in Kcal/mol). D and H Time percentage of hydrogen bonds. An i-j residue pair was defined as in contact when an atom in the ith residue and an atom in the jth residue were closer than 4 Å and j>i+2. (TIF) [file pcbi.1003249.s012.tif]

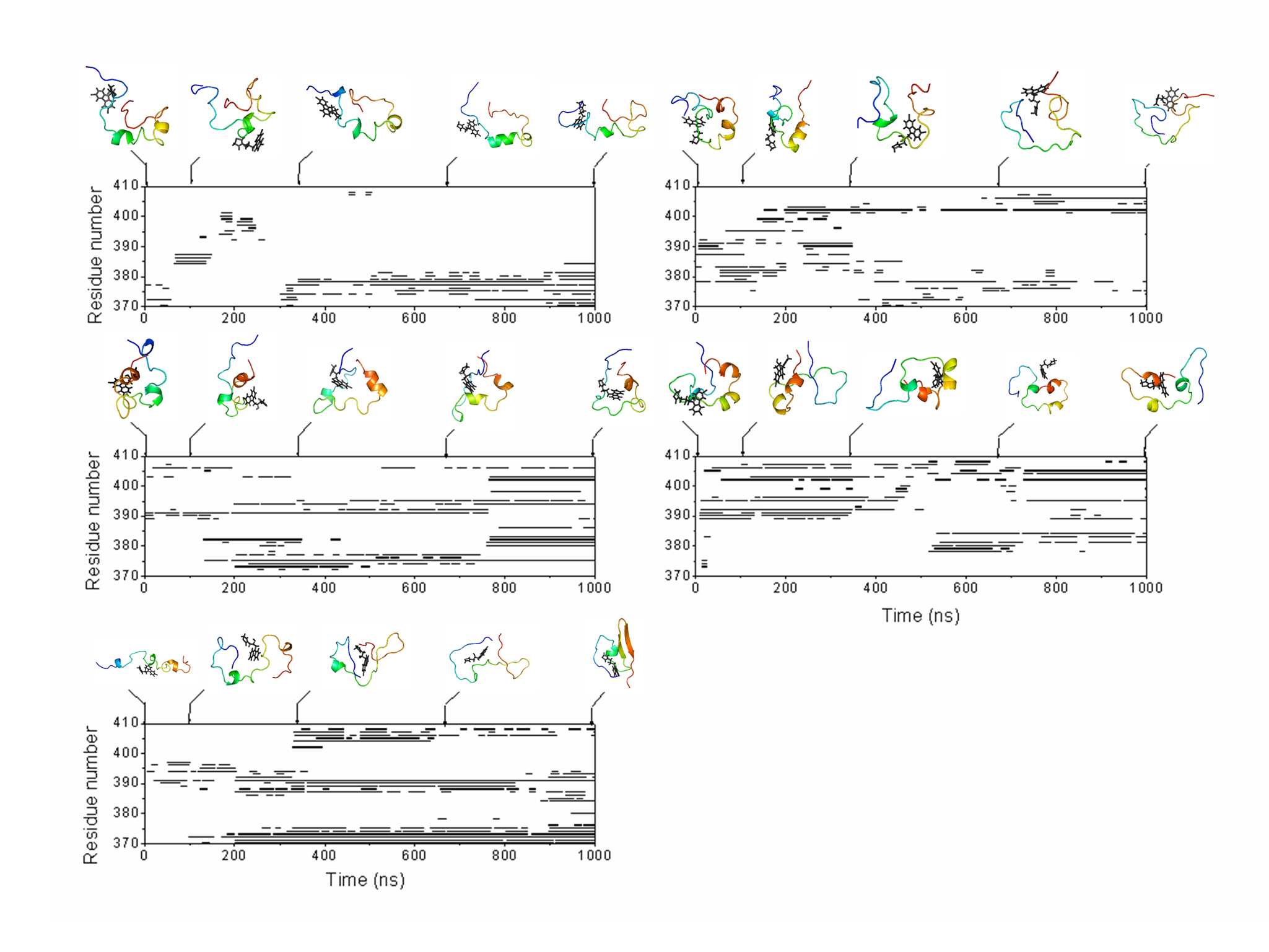

Supplement: Figure S13 — Binding sites of holo c-Myc370–409 determined by ΔSASA as a function of time for five MD trajectories with explicit solvent simulations. Binding residues were defined by ΔSASA larger than 10 Å2 and are shown in squares. Continuous binding of less than 10 ns was ignored. (TIF) [file pcbi.1003249.s013.tif]

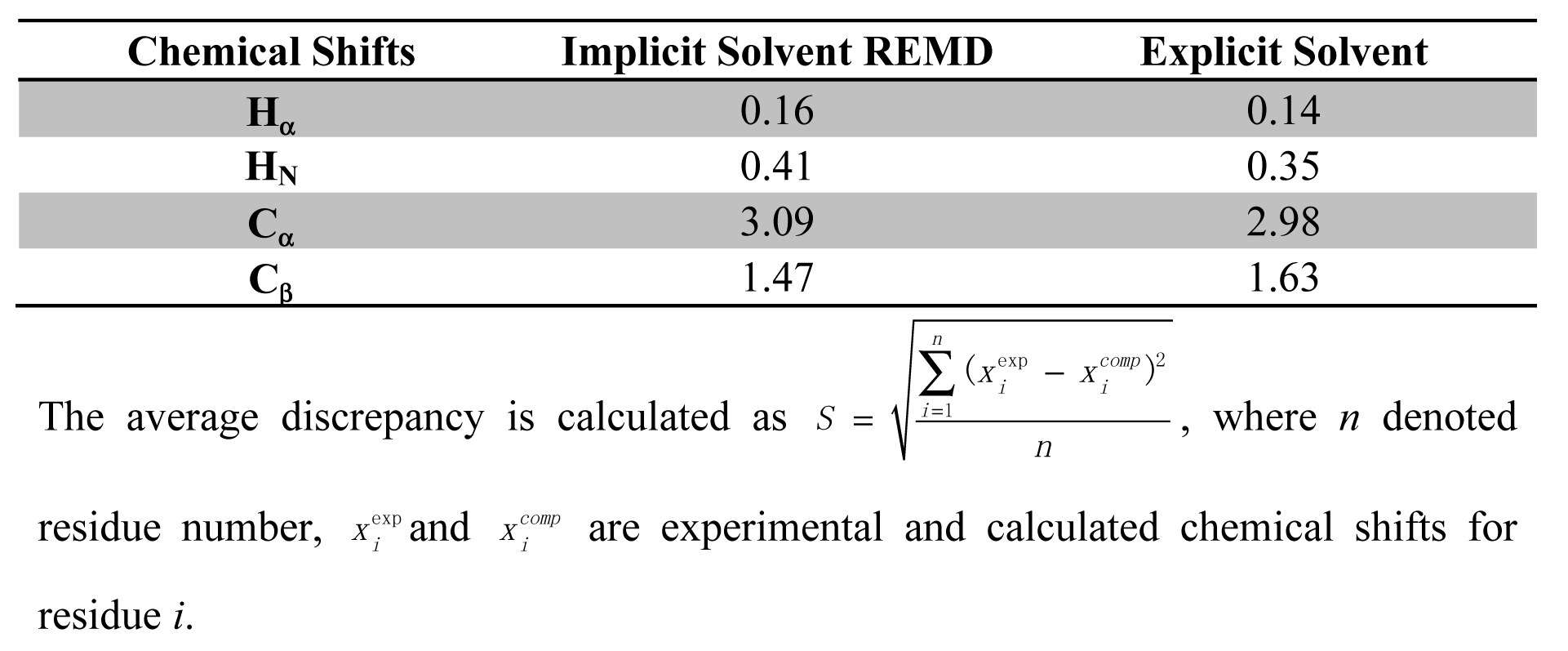

Supplement: Table S1 — Average discrepancy between simulated and experimental chemical shifts for Hα atoms of apo c-Myc370–409 calculated using SHIFTS. (TIF) [file pcbi.1003249.s014.tif]
